# On the origin of life in the Zinc world: 1. Photosynthesizing, porous edifices built of hydrothermally precipitated zinc sulfide as cradles of life on Earth

**DOI:** 10.1186/1745-6150-4-26

**Published:** 2009-08-24

**Authors:** Armen Y Mulkidjanian

**Affiliations:** 1 School of Physics, Universität Osnabrück, D-49069 Osnabrück, Germany; 2 A.N. Belozersky Institute of Physico-Chemical Biology, Moscow State University, Moscow, 119991, Russia

## Abstract

**Background:**

The complexity of the problem of the origin of life has spawned a large number of possible evolutionary scenarios. Their number, however, can be dramatically reduced by the simultaneous consideration of various bioenergetic, physical, and geological constraints.

**Results:**

This work puts forward an evolutionary scenario that satisfies the known constraints by proposing that life on Earth emerged, powered by UV-rich solar radiation, at photosynthetically active porous edifices made of precipitated zinc sulfide (ZnS) similar to those found around modern deep-sea hydrothermal vents. Under the high pressure of the primeval, carbon dioxide-dominated atmosphere ZnS could precipitate at the surface of the first continents, within reach of solar light. It is suggested that the ZnS surfaces (1) used the solar radiation to drive carbon dioxide reduction, yielding the building blocks for the first biopolymers, (2) served as templates for the synthesis of longer biopolymers from simpler building blocks, and (3) prevented the first biopolymers from photo-dissociation, by absorbing from them the excess radiation. In addition, the UV light may have favoured the selective enrichment of photostable, RNA-like polymers. Falsification tests of this hypothesis are described in the accompanying article (A.Y. Mulkidjanian, M.Y. Galperin, *Biology Direct *2009, 4:27).

**Conclusion:**

The suggested "Zn world" scenario identifies the geological conditions under which photosynthesizing ZnS edifices of hydrothermal origin could emerge and persist on primordial Earth, includes a mechanism of the transient storage and utilization of solar light for the production of diverse organic compounds, and identifies the driving forces and selective factors that could have promoted the transition from the first simple, photostable polymers to more complex living organisms.

**Reviewers:**

This paper was reviewed by Arcady Mushegian, Simon Silver (nominated by Arcady Mushegian), Antoine Danchin (nominated by Eugene Koonin) and Dieter Braun (nominated by Sergey Maslov).

## Background

- ....*without parent by spontaneous birth*

*Rise the first specks of animated earth*.

Erasmus Darwin, *Temple of Nature*, 1802 [1]

The problem of the origin of life is central to biology. It has been repeatedly addressed by scholars, including the above-quoted Erasmus Darwin and his famous grandson Charles, who wrote in his letter to J.D. Hooker of February 1, 1871: "*It is often said that all the conditions for the first production of a living organism are now present, which could ever have been present. But if (and oh! what a big if!) we could conceive in some warm little pond, with all sorts of ammonia and phosphoric salts, light, heat, electricity, &c., present, that a proteine [sic] compound was chemically formed ready to undergo still more complex changes, at the present day such matter would be instantly devoured or absorbed, which would not have been the case before living creatures were formed*" [2]. Fifty years later, Oparin has suggested, in the first comprehensive scenario of the abiogenic origin of life (abiogenesis), that the primordial reducing atmosphere could have favoured the *spontaneous *formation of proteinaceous bodies that could aggregate into coacervates (protocells) [3,4]. Independently, Haldane, building upon the achievements of virology, proposed that the life started from bacteriophage-like molecules synthesized under the influence of the Sun's radiation in the primordial "hot dilute soup" [5]. It has been repeatedly demonstrated [6-17] that simple building blocks such as amino acids or nucleobases could form from simpler compounds, provided that energy was delivered as UV light or electric discharges (see [18-36] for surveys of research on the origin of life, and a section below devoted to the more detailed consideration of particular concepts).

The initial, rather general Oparin-Haldane's concept of abiogenesis has been gradually replaced by a mosaic of specific hypotheses that either emphasize the "replication first" principle or build upon the "metabolism first" assumption. The "replication first" concept implies that the emergence of the first replicating entities (replicators) preceded metabolism; it is represented by the RNA World scenario that implies that RNA-like molecules capable of both self-reproduction and simple metabolism were the first inhabitants of Earth [37-73]. The "metabolism first" idea suggests that life started as a system of interacting chemical cycles and the first replicators appeared later, see refs. [23,27,29,30,36,74-84] for consideration of the controversy between the two concepts. Elsewhere, we have argued that the "replication first" and "metabolism first" concepts complement rather than contradict each other and have suggested that life on Earth started with a "metabolism-driven replication" [85]. We have also emphasized that the virtually unlimited number of tentative scenarios of the origin of life can be dramatically reduced by the simultaneous consideration of a variety of external constraints (boundary conditions) [85].

Here I invoke further (bio)energetic, physical, and geological constraints that are related to abiogenesis. As a solution that satisfies these constraints, I put forward an evolutionary scenario in which life on Earth emerged, powered by solar irradiation, within porous edifices of hydrothermal origin that were built of photosynthesizing zinc sulfide (ZnS) crystals, in the "Zn world".

### Energetic, physical, and geological constraints on abiogenesis

#### Energetics: Requirement for utilizable energy flow(s)

Living organisms can exist only when supported by energy flow [86-92]. Because of the obvious requirement for energetic continuity, the energy flows that deserve attention in an evolutionary context are those that remain constant on the evolutionary relevant, geological timescale. This consideration discounts the evolutionary importance of occasional energy inputs such as impact bombardment, atmospheric electric discharges, and shock waves. The primordial atmosphere on Earth is assumed to be dominated by carbon dioxide [25,93-100]. Hence, energy was initially needed to reduce CO_2 _to organic compounds that could participate in prebiological syntheses [34]. Currently, the fixation of CO_2 _by living organisms is supported by two energy fluxes: the communities at the Earth's surface depend, via photosynthesis and its products, on solar light [101], whereas the biotopes at the sea floor can also exploit the redox potential difference between the reduced hydrothermal fluids and oxygenated ocean waters [102]. Accordingly, some scholars have considered solar radiation to be the driving force of abiogenesis [5,85,103-112]. Others have hypothesized that chemical or redox disequilibria at the sea-floor hydrothermal vents [113-123] or at the surface of sea-floor iron minerals [124-130] could have driven the emergence of the first organisms. As argued in more detail elsewhere [85], a direct analogy between primordial life and modern deep-sea biotopes is not possible, since the redox energy span of > 1 eV between the reduced compounds of hydrothermal fluids and the sea-dissolved oxygen became exploitable only after the ocean waters – only 2 Ga ago – were saturated by oxygen, a by-product of cyanobacterial photosynthesis [101,131,132].

The very lack of oxygen in the primordial atmosphere should, however, favour light-driven chemical syntheses. Without the ozone shield, the solar light reaching Earth contained a UV component that was 10–1000 times stronger than it is today [133,134] and could have driven diverse chemical reactions, in particular carbon fixation. The major constituents of the primordial atmosphere (CO_2_, N_2_, CH_4_, and water vapour [25,93-100]) let UV rays with λ > 240 nm through [133]. The fossils of phototropic communities, which apparently flourished as far back as 3.4–3.5 Ga [25,135-139], also indicate that the primordial atmosphere was transparent to solar light. Hence, no other known energy source could compete with solar irradiation in terms of strength and access to the whole of the Earth's surface.

Mauzerall has introduced an important additional constraint by noting that the energy requirements of the first living beings had to be compatible with those of modern organisms [109]. He argues that "*the ur-cell would be simpler, but it would also be less efficient*". More rigorously speaking, the intensity of the energy flux(es) that supported the emergence of life should be either comparable with the intensity of modern life-supporting energy flows or stronger. At least two UV-driven abiogenic processes of CO_2 _reduction are known to proceed with an efficiency comparable to that of modern photosynthesis. On the one hand, the photo-oxidation of ferrous iron ions in solution can lead to the reduction of CO_2 _[10]; for example, Borowska and Mauzerall have observed a light-driven formaldehyde formation in the presence of dissolved ferrous hydroxide with a quantum yield of 2–3% [140]. On other hand, a UV-driven synthesis of diverse organic compounds from CO_2 _has been demonstrated at the surface of broad-band semiconductors [141-149]. Such semiconductors not only photoreduce CO_2 _but, depending on the initial substrates, can also photocatalyse a wide set of diverse organic reactions [144,150-152]. Several naturally occurring minerals, in particular TiO_2 _(anatase/rutile), WO_3 _(wolframite), MnS (alabandite), and ZnS (wurtzite, sphalerite), possess the properties of broad-band semiconductors and can photoreduce CO_2 _[107,143,151,153-158]. The highest quantum yield of 80% has so far been reported for CO_2 _reduction to formate at the surface of colloidal ZnS particles [144,145].

#### Physics: Photostability of nucleotides

RNA and DNA are polymers of similar sugar-phosphate units, with each sugar moiety (ribose in RNA or deoxyribose in DNA) carrying one of four different nitrogen bases (nucleobases). The specific feature that is shared by all nucleobases is their unique photostability [159-165]. Since this trait is not related to the storage of genetic information, several authors [105,112,133,159,164,165] have noted that this property could have been of some use when the UV flux at the surface of primordial Earth was much stronger than it is now [133,134]. Nucleobases apparently can absorb excess energy quanta from sugar-phosphate moieties and protect them from photo-dissociation [166]. This feature explains why the UV damage to the backbones even of modern RNA and DNA molecules is 10^3^–10^4 ^times less frequent than destruction of nucleobases themselves [159].

Based on a Monte-Carlo simulation of primordial photochemistry [112], we have proposed an evolutionary scenario in which the relative enrichment in increasingly complex RNA-like polymers could be attributed to their higher photostability in a UV-irradiated environment, with UV-quenching nucleobases protecting the sugar-phosphate backbones from photo-dissociation. It was posited that the photostability could increase further owing to the stacking of nucleobases and the formation of Watson-Crick pairs [85,112], see also below.

In modern organisms, the continuous victimization of nucleobases is a well-known problem that is counteracted by sophisticated repair systems [167]. At the earliest steps of evolution, repair systems were absent, so the photodestruction of nucleobases could have hindered the selection of the first replicators. The photodestruction of nucleobases could be, however, prevented by radiation-absorbing templates. Many minerals can take up radiation energy from the adsorbed photoactive compounds. For example, montmorillonite particles have been shown to protect catalytic RNA molecules (hairpin ribozyme 1) from UV-induced damage: after a UV-irradiation, the self-cleavage activity of the montmorillonite-adsorbed ribozyme molecules was three times higher compared to that of the molecules irradiated in the absence of montmorillonite [168]. With ZnS crystals, the excitation transfer from adsorbed dye molecules to a template has been shown to proceed within picoseconds [169], i.e. much faster than the typical intrinsic characteristic time of photodestruction (e.g. ~20 μs for adenosine monophosphate [170]). Hence, in the evolutionary context, the first photostable RNA-like polymers had better survival chances at the surfaces of those minerals that could efficiently absorb the radiation energy.

#### Geology: Requirement for hydrothermal settings

In addition to abundant chemical elements such as carbon, oxygen, nitrogen, and hydrogen, biological systems contain a number of microelements, often at levels far exceeding those in the surrounding environment (see [171] for a comprehensive survey). In particular, transition metals are often involved in enzyme catalytic centres [90,172]. The concentration of such metals in modern cells is many orders of magnitude larger than that in sea water (see Table 1); the ion accumulation is accomplished by sophisticated transport systems and demands ion-tight membranes to prevent the escape of trapped metal ions out of the cell [173,174]. However, the ion-tight membranes, as argued elsewhere [175,176], seem to be a relatively late evolutionary acquisition. Here we encounter a paradox. On the one hand, the emergence of metal-containing RNA and protein domains – as a result of their eventual stabilization by available transition metal ions – implies an abundance of these ions. On the other hand, the *equilibrium *concentration of such ions in sea water is very low (see Table 1). This paradox is routinely resolved by invoking hydrothermal settings as potential cradles of life [113-123,177]. In such systems, which currently cluster around the mid-ocean ridges and deep-sea submerged volcanoes (seamounts) – where hot magma chambers occur near the seabed – water circulates down into the crust, becomes heated, and then rises up. When water is overheated to more than 400°C, it can leach metal ions from the crust. These ions are then brought to the surface by hot hydrothermal fluids, so that the steady-state concentrations of metal ions at the orifices of hydrothermal vents may exceed the equilibrium concentrations because of this continuous supply [102,178].

**Table 1:** Approximate total concentration of key ions in various environments (in moles/litre).

Ion	Modern sea water	Blood plasma	Cell cytoplasm
Na^+^	0.4	0.14	0.01

K^+^	0.01	0.005	0.1

Ca^2+^	0.01	0.002	0.001

Mg^2+^	0.05	0.001	0.01

Fe	10^-8 ^(mostly Fe^3+^)	10^-5^	10^-3^–10^-4^

Mn^2+^	10^-8^	10^-8^	10^-6^

Zn^2+^	10^-9^	10^-5^	10^-3^–10^-4^

Cu	10^-9 ^(Cu^2+^)	10^-5^	10^-5^

Cl^-^	0.5	0.1	0.1

The table is compiled from data presented in [90,102,172,173].

Since hydrothermal fluids are rich in H_2_S, the interaction of metal-rich hot hydrothermal fluids with cold ocean water leads to the precipitation of metal sulfide particles that form "smoke" over the "chimneys" of deep-sea hydrothermal vents [102,178]. These particles eventually aggregate, settle down, and, ultimately, form porous, sponge-like structures around the vent orifices [179-181]. The vent systems have a zonal structure [102,178,182]: pyrite (FeS_2_) and chalcopyrite (CuFeS_2_) are found in the centre, where the temperature of hydrothermal fluids is the highest (~350°C; the water at the sea floor remains liquid even at such high temperatures because the pressure is above 200 bar [102]). At the periphery of hydrothermal fields, the temperature of hydrothermal fluids is lower because the rising hot fluids mix, while still under the sea floor, with the cold ocean waters that are pressed into the seabed by the overlying water column. Those peripheral chimneys that eject fluids with temperature in the range of 200°C to 300°C are covered by porous precipitates of sphalerite (ZnS), with additions of other sulfides such as galena (PbS) and alabandite (MnS) [102,180,182]. This change in chemical composition is due to the fact that upon cooling, the sulfides of iron and copper precipitate much faster than those of zinc and manganese [183]. Accordingly, when the temperature of hydrothermal fluid is less than 300°C, the sulfides of iron and copper precipitate already below the sea floor, inside the moulds of hydrothermal systems, so that the transition metal ions that reach the surface are predominantly Zn^2+ ^[184]. The difference in the precipitation rates manifests itself also in the chemical composition of those vent chimneys that eject both Fe^2+ ^and Zn^2+ ^ions. The throats of such chimneys are formed of promptly precipitating FeS and CuFeS_2_, while their outer surfaces are coated by the more slowly precipitating ZnS [179,181].

The zonal structure is remarkably conserved between the modern hydrothermal vent systems and the ancient volcanogenic massive sulfide (VMS) deposits of hydrothermal origin. VMS deposits can reach many kilometres in diameter, and date back to the Archean period [185-187]. The ancient VMS deposits have pyrite (FeS_2_) and chalcopyrite (CuFeS_2_) at their centres being encircled by consecutive halos of, e.g., pyrite-chalcopyrite-sphalerite, sphalerite-galena-alabandite, and, finally, chert [185,187].

#### ZnS crystals: Unique traits

The above listed constraints, when considered simultaneously, identify crystalline ZnS as the single compound that (i) can serve as an efficient photocatalyst capable of reducing CO_2 _with a quantum yield of up to 80%, (ii) can promptly absorb UV quanta from the adsorbed organic compounds, preventing their destruction, and (iii) is a major constituent of hydrothermal vent systems, being typically found at their outer surface and/or periphery.

The evolutionary scenario that is given below suggests that porous ZnS formations of hydrothermal/volcanic origin performed several functions, being involved in the primeval photosynthesis of the first metabolites, in the (photo)selection of the first RNA-like polymers, and in their protection from photodestruction.

## Hypothesis: Emergence of the first biopolymers at photosynthesizing ZnS edifices of hydrothermal origin

*IN earth, sea, air, around, below, above*,

Life's subtle woof in Nature's loom is wove;

*Points glued to points a living line extends*,

Touch'd by some goad approach the bending ends;

*Rings join to rings*...

Erasmus Darwin, *The Temple of Nature*, 1802 [1]

### Initial geological settings

**Figure 1:**
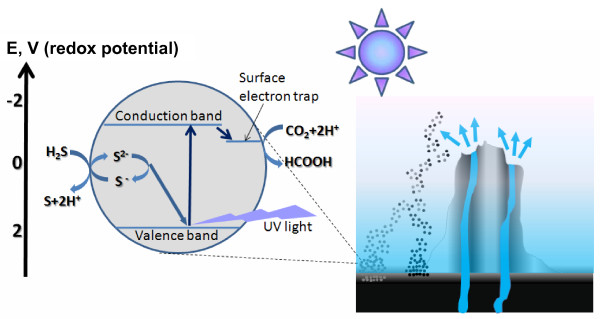
**Primeval ZnS-mediated photosynthesis in sub-aerial, illuminated settings**. **Right**: Precipitation of FeS and ZnS nanoparticles (black and grey spots, respectively) around a primeval, sub-aerial hot spring. Note that ZnS and FeS particles precipitate at different distances from the spring. The picture is based upon data from [102,119,179,181,183]; see the main text for further details. **Left**: A schematic presentation of reactions within a photosynthesizing ZnS nanoparticle, as combined with an energy diagram; the scheme is based on refs. [145,149,190,278]. Initially the absorption of a UV quantum leads to the separation of electric charges. The electrons migrate in the crystal until they are trapped at the surface; the trapped electrons can reduce a CO_2 _molecule either via two one-electron transfers [144] or, possibly, in a concerted two-electron reaction. The electron vacancy (hole) is initially reduced by the S^2- ^ion of the crystal; the ultimate electron equilibration, as discussed in the main text, requires external electron donors, e.g. H_2_S. Note that, for simplicity of presentation, the one-electron and two-electron reactions are not discerned; see the main text for further details.

After the primary hydrogen atmosphere of Earth had escaped into space, the so-called secondary atmosphere built up with volcanic gases; this atmosphere was, most likely, dominated by CO_2_, with smaller amounts of N_2_, CO, and H_2_, similar to that on modern Mars and Venus, where CO_2 _still makes up 95% of the atmosphere [25,93-100]. As the Earth's surface gradually cooled, water vapour started to condense into the first oceans. The atmospheric pressure at the surface of primordial Earth has been estimated to reach several hundred bars; therefore ocean formation could have started when the surface was still very hot [94,97,100]. Zircon data indicate the presence of the first continent(s) by 4.2 Ga [188]. Hydrothermal activity of some type would have been established promptly, driven by thermal convection. Most likely, the initial convection systems did not form a continuous chain of mid-ocean ridges as they do now but a pattern of "hot spots" similar to modern volcanic island arcs [93,95,189]. The activity of these hydrothermal/volcanic systems was accompanied by surges of hot hydrothermal fluids to the surface. Owing to the initial atmospheric pressure of ≥ 100 bar (see [94,100], and *cf*. with the pressure of 95 bar at the surface of modern Venus), very hot hydrothermal fluids enriched by dissolved metals could discharge directly to the surface of the first continents. This situation differed fundamentally from the modern one, since today such hot fluids can reach the continental surface only as steam (at the points of volcanic or geyser activity), losing their metal content on the way. Taking into account the slow precipitation of ZnS under high-pressure conditions [183] and the abundance of Zn in the Earth's crust [90], one can expect a major delivery of Zn-enriched hot fluids to the surface of the first continent(s). Zinc could even have been the dominant transition metal in the continental hydrothermal fluids when the atmospheric pressure changed in the range from ca. 100 bar to ca. 10 bar (this would correspond to the temperature of hot fluids reaching 300°C – 200°C, respectively, *cf*. with the above described situation at modern hydrothermal vents). Hence, it is possible that the large areas of first continent(s) could have been covered by porous ZnS precipitates of hydrothermal origin. These ZnS edifices should have been accessible to solar UV radiation at the surface of continents and in shallow waters surrounding them (see Fig. 1). Hereafter, the term "sub-aerial" is used to denote illuminated settings where the UV-rich solar light could have served as an energy source for primordial syntheses (see also [25,114,177]).

### ZnS-mediated photosynthesis

In the absence of an ozone layer, the UV component of solar radiation would have driven the reduction of CO_2 _at the ZnS-covered surfaces. Since these surfaces were formed by precipitated ZnS particles (see Fig. 1), similar to those used in the aforementioned experiments with the photosynthetically active "colloidal" ZnS crystals [144,145], the reduction of CO_2 _may have proceeded with a high quantum yield under the high atmospheric CO_2 _pressure. Zinc sulfide is a very powerful photocatalyst that, besides reducing CO_2_, is capable of driving diverse reactions of carbon- and nitrogen-containing substrates [144,150,152,190-192]. Such substrates could build up in the atmosphere, be generated by photochemical reactions in the water phase [24,106,109], accompany volcanic extrusions [193] and hydrothermal fluids [123], or be brought by meteorites [194]. They could have then participated in further photocatalyzed transformations at the ZnS surfaces.

### First settlers in the ZnS world

Electrically charged products of photosynthesis, e.g. negatively charged carbonic acids, would have been attracted by the complementary charges at the ZnS surfaces of mineral compartments. These molecules could have interacted with each other at the catalytic surfaces of continuously operating porous ZnS photoreactors, yielding even more intricate carbon- and nitrogen-containing molecules. The more complex molecules would, generally, have absorbed more light and been more vulnerable to UV quanta. Still, in certain cases, the increase in chemical complexity may have been accompanied by an increase in photostability. Indeed, the destruction of a chemical compound by a light quantum starts with the "trapping" of its energy by a particular chemical bond, followed by an increase in the energy of this bond and its eventual dissociation [195]. However, if the absorbed energy is spread over many bonds, then the probability of bond cleavage drops dramatically. Such a spreading of excitation energy occurs in systems that contain conjugated bonds (so-called π-systems with alternating single and double bonds); the spreading is facilitated by a ring-like (aromatic) molecular structure. All nucleobases belong to such ring-like, conjugated systems [159]; the lifetime of their excited states (~100 fs [164]) is extremely short even for π-systems; this short life time would additionally have decreased the probability of photodestruction. As discussed above, the UV-resistance of π-systems could increase further upon their stacking together and/or adsorption to radiation-absorbing minerals.

Hence, at illuminated ZnS surfaces, the UV-resistant, ring-like compounds could survive as stacked aggregates of "rings joined to rings" (quoted from [1]). A relative enrichment in such stacks, as well as their stabilization by covalent linkages, could be driven by a number of factors, namely, the UV-resistance of polymerized *and *stacked π-systems [112,164], the potential to utilize the energy of UV quanta for photopolymerization [11,12,196], the ability of Zn^2+ ^ions to catalyze the polymerization [197,198], and the low dielectric permittivity of the surface-adjoining water layers [199] that may have favoured condensation reactions. In addition, a regular mesh of electric charges at the ZnS surface, by attracting reactants and arranging them appropriately relative to each other, may have made the polymerization thermodynamically more favourable than in bulk water [124]. Then, however, the resulting polymers should have stayed confined to the surface [200]. In the context of a UV-irradiated environment such confinement could be considered a rescue since an eventual detachment of a primordial polymer from the energy-absorbing ZnS surface would have led to faster photodestruction. At the same time, while prevented from detachment from the surface, the molecules would have been able to diffuse along the surface, to interact with each other, and to form aggregates, which is a pre-condition for the abiogenic emergence of increasingly complex structures.

The scenario outlined above should yield two fundamentally different populations of molecules, namely surface-confined, relatively complex structures capable of efficient discarding excess radiation energy (hereafter referred to as *zymes*), and continuously photosynthesized simpler organic molecules – the future *metabolites *– that stored the solar energy in their covalent bonds.

### First replicating entities

The eventual elongation of zymes would promote their entropy-driven folding at the surface. In addition, an increase in their amount would encourage interactions between them. Both factors may have led to the formation of hydrogen bonds within zymes and/or between different zymes. Clustering of zymes may have been favoured by high pressure [201] and periodic drying events, e.g. in tidal regions [18,202], resulting in a kind of natural polymerase chain reaction (PCR)-like process [203]. The UV-stability of double RNA strands, owing to hydrogen bonding, is much higher than that of single-stranded RNAs [159,164,204-206]. Therefore those π-systems may have been selected – from the initially larger set of compounds – which could make multiple hydrogen bonds with each other. Ultimately, this selection would have led to a relative enrichment of complementary nucleobases, including those that we know now. It is plausible that the π-systems could have been initially linked in various ways. It would appear that one of these constructs, the one with ribose-phosphate units connecting the stacked nucleobases, attained the ability for self-replication and was, because of this, retained by evolution.

Unlike occasional self-replication events, a systematic, accurate replication is thermodynamically demanding [207] and would require special machinery that could consist of several folded polymers resembling modern transfer RNA (tRNA) or ribosomal RNA (rRNA). It is unlikely that we would ever be able to reconstruct all the steps that led to the formation of first replicators (see but refs. [44,52,54,58,60,65,67,72,208,209] for tentative scenarios). We can, however, try to make guesses on the selective forces underlying their emergence. At least two factors deserve note:

1) Nucleobases quench the UV quanta by converting the energy into heat. The heating, however, is not harmless. Using the approach of Dancshazy and co-workers [210], it is possible to surmise that a single UV quantum should locally heat a 100-unit RNA-like polymer by tens of degrees Celsius. Even if the absorbed energy can be promptly transmitted to a template, local heating of the template can eventually cause ablation of the molecule [211]. In the UV-irradiated environment, both outcomes could lead to polymer deterioration. Overheating, however, can be avoided by channelling part of the energy into work. For example, those zymes that could serve as antennas and use the UV energy, e.g., for connecting nucleotides together (as hypothesized by Skulachev [111]) had better survival chances. This selective advantage of "working" polymers over the "idle" ones is general in nature: it is applicable not only to the first ribozymes, but also to the first proteins, as discussed in the next section.

2) A population of RNA-like polymers adsorbed at a ZnS template could gather light and therefore enhance the yield of the photocatalysis. Since the hydrothermal ZnS structures are highly porous [179-181], the pores/compartments that contained efficient replicators and, hence, an increasing number of UV-absorbing zymes could produce more metabolites, which, in turn, could be used to build new replicators, resulting in a kind of positive feedback.

As first pointed out by Horowitz [212], and as our simulations showed ([112]; see also the section on energetics of the Zn world below), an abiogenic formation of complex molecules (e.g. long polymers) would imply the presence of an overwhelmingly larger amount of simpler molecules of the same type (shorter polymers). The first replicators could therefore utilize the available shorter fragments; with time, they could acquire the ability to attain building blocks by cleaving the (phosphor)ester bonds in other surface-adsorbed zymes. Owing to this development: (i) a coupling mechanism could be established that, with some modifications, has been in use ever since – the polymerization of RNA and DNA is still driven by cleavage of the phosphoester bonds in ATP; and (ii) the genuine fight for survival could begin, since each replicator became a potential prey for others.

### Emergence of proteins and enzymes

Generally, the thermodynamics of primordial syntheses of polymers, and in particular of polypeptides, has been deemed a riddle since the respective condensation reactions, which are accompanied by the release of water molecules, should be unfavourable in water (see e.g. refs. [83,213] for further discussion of this point). A closer inspection of modern metabolic chains offers a way out of this conundrum. Although biological syntheses are indeed accompanied by the release of water molecules, they never proceed "for free", but are coupled to thermodynamically favourable, exergonic reactions, e.g. of ATP hydrolysis, which require water. Hence, if (i) there were substrates that could be hydrolyzed and (ii) a thermodynamic coupling between syntheses and hydrolyses could be established – then the primordial syntheses could proceed without violating the laws of thermodynamics.

In this framework, the emergence of new synthetic pathways could proceed in two steps, as follows. First, a (ribo)zyme capable of cleaving a new type of chemical bond – in a thermodynamically favourable reaction – would have emerged. Then, the ability to *make *this particular type of chemical bond could develop as a reversal of the new catalytic pathway, provided that coupling with some exergonic reaction (e.g. a phosphate group transfer or a hydrolysis of a phosphoester bond) could be established.

In the ZnS settings, photosynthetically produced polycarbonic compounds, inorganic polyphosphates, RNA-like oligomers, and diverse low-molecular-weight phosphorylated derivates could serve as cleavage substrates for the first synthases. The reducing equivalents that would be needed for some synthetic reactions were continuously generated at the illuminated ZnS surfaces; nucleotide-containing redox cofactors, such as NADH, NADPH, FAD, and FMN [22,214-216], could have emerged as mediators that picked up the photoexcited electrons from photoactive ZnS surfaces and delivered them to the respective ribozymes.

After the emergence of the first replicators capable of connecting amino acids by peptide bonds, the synthesis of first, random polypeptide sequences at nucleotide and/or ZnS templates could begin. Without considering the elusive chemical details, it seems fitting, as in the previous section, to focus on selective factors that could drive the emergence of the first polypeptides. They may have been recruited to perform functions that did not require a particular amino acid sequence but only the ability of a polypeptide to bind to a replicator, e.g. via the protein backbone groups [209]. Such a binding, for example, could protect the backbone of replicators from hydrolysis or cleavage. In addition, the bound polypeptides could absorb heat into which the energy of UV quanta was converted, therefore protecting the replicators from thermal damage. It is noteworthy that, if a protecting polypeptide could eventually discard excess energy by funnelling it into a chemical reaction, then the probability of denaturation would have become lower. Those replicators that could synthesize proteins capable of supporting catalysis by (ribo)zymes [67] or catalyzing useful chemical reactions themselves may have got an advantage. Eventually such a selection could establish a correspondence between the nucleotide and amino acid sequences, see [46,47,53,58,67,73,217,218] for tentative scenarios on the evolution of genetic code and protein synthesis.

### The first colonization wave

The ZnS precipitates would have attenuated the UV component of solar light, thus providing shade for the inhabitants of the lower-lying compartments. Hence a stratified system could be established with the illuminated upper layers accounting for maximal photosynthesis, and lower, less productive, but more protected layers providing shelter for the first replicating entities in their pores. The porosity of the ZnS precipitates [179,180] would have enabled metabolite transport between the layers. Moreover, the sponge-like inner structure could eventually enable variable hydration of the compartments that may have served as an additional selective factor. Both the gradient of light and the interlayer metabolite exchange are typical of modern stratified, seashore phototrophic communities (e.g. stromatolites [25,93,139]).

Those consortia of replicators that were able to couple useful syntheses with exergonic chemical reactions could have became relatively independent of upper, photosynthesizing layers and could penetrate the depths of their porous habitats. This penetration can be considered the first wave of the Earth's colonization by living organisms that eventually evolved from replicating zymes. This colonization would have been supported by exchanges of metabolites between the illuminated strata and those that were deeper and darker. In the darkness, direct contact between the RNA-like "bodies" of replicators and the radiation-absorbing ZnS template was no longer crucial, so that the replicators could evolve into life forms that were enclosed in protein envelopes resembling modern viruses [62,70,219].

The energetics of these communities consisted in the interplay between the continuous ZnS-mediated photosynthesis and the increasingly complex heterotrophy of the first organisms. This heterotrophy may have been based on coupling the exergonic breakdown of photosynthetically produced metabolites with endergonic (bio)synthetic reactions. This same interplay of photosynthesis and heterotrophy still drives the majority of terrestrial communities today.

### The fall of the Zn world

Upon further decrease in the amount of CO_2 _in the atmosphere and, accordingly, in atmospheric pressure, the delivery of Zn-rich fluids to the surface of continents would have gradually ceased, so that fresh, photosynthetically active ZnS surfaces could no longer form in sub-aerial settings. After that, ZnS-rich hydrothermal edifices could persist only deeply at the sea floor, ultimately clustering around the mid-ocean ridges. The organisms that remained confined to the sub-aerial settings would have found alternative ways to reduce CO_2_. Moreover, in the absence of a zinc supply, they were forced to confront unfamiliar minerals, in particular those containing iron. At that time the dominating transition metal ion in sea water would have been Fe^2+ ^[90,220,221]. Iron, unlike zinc, is redox-active and can generate harmful hydroxyl radicals [222-225]. It would seem that the expatriates of the Zn world had to be full-fledged organisms with reliable replication machinery, robust metabolism, and protective envelopes. The story of how they could populate the Earth is beyond the problem of the origin of life proper and hence remains out of the scope of this communication; this topic, however, is addressed in the accompanying article [226].

### Validation of the hypothesis

*First forms minute, unseen by spheric glass*,

Move on the mud, or pierce the watery mass;

These, as successive generations bloom

*New powers acquire and larger limbs assume*.

Erasmus Darwin, *The Temple of Nature*, 1802 [1]

The search for a solution to the origin of life puzzle is hindered by the impossibility of providing an ultimate experimental proof, namely to (re)produce life from scratch *de novo *(see e.g. [58,218]). In view of this obstacle, Wächtershäuser [227] has suggested that the hypotheses on the origin of life could be validated by rigorous application of Karl Popper's principles of testing scientific theories [228,229]. Popper wrote: "*We may if we like distinguish four different lines along which the testing of a theory could be carried out. First there is the logical comparison of the conclusions among themselves, by which the internal consistency of the system is tested. Secondly, there is the investigation of the logical form of the theory, with the object of determining whether it has the character of an empirical or scientific theory, or whether it is, for example, tautological. Thirdly, there is the comparison with other theories, chiefly with the aim of determining whether the theory would constitute a scientific advance should it survive our various tests. And finally, there is the testing of the theory by way of empirical applications of the conclusions which can be derived from it*" (quoted from ref. [229]). The core of Popper's approach is the idea of falsification, i.e. putting a hypothesis to the test by making testable predictions and checking them. The falsification tests of the Zn world hypothesis, because of their key importance, are described in a separate accompanying article [226]. Here I consider only the empirical supporting evidence, the internal consistency of the hypothesis, and the relation of the posited concept to other hypotheses on the origin of life.

### Supporting evidence

Below, the supporting data are organized along the steps of the above-presented evolutionary scenario. One outstanding feature, however, deserves preferential treatment since it is crucial for almost every one of these steps. This feature is the unique ability of ZnS crystals to store radiation energy (see [144,230-233] for reviews). This property manifests itself in phosphorescence (afterglow), so that ZnS – widely known as "phosphor" – is used in numerous devices, from various types of displays to 'glow in the dark' items. ZnS is a broad-band *n*-type semiconductor with a gap band energy of 3.2–3.9 eV (depending on the particular crystal structure). When radiation strikes such a semiconductor, it may excite an electron and consequently leave a "hole" (see Fig. 1). If the energy of the radiation quantum is larger than the band gap energy, the electron reaches the so-called conduction zone and can move inside the crystal. Ultimately, the electron can recombine with the hole. However, if the electron gets into an energy trap (see Fig. 1), then the recombination can proceed only slowly, under the condition of thermal activation [234]. In pure ZnS, the atoms at the surface can trap electrons [234,235], particularly if the semiconductor interacts with a polar solvent such as water. At the surface, a photoexcited electron loses part of its energy owing to the interaction with the molecules of the solvent and, accordingly, is prevented from returning into the bulk of ZnS. In addition, recombination may be prevented by prompt replenishing the hole with an electron coming from a potent electron donor ("hole scavenger"). The trapped electron then remains confined to the surface until an external electron acceptor, e.g. a molecule of CO_2_, picks the electron up, see [144,190,234,236] for reviews.

Small nanocrystals of ZnS and of its chemical "twin" CdS have been found to behave as quantum dots – systems with physical properties intermediate between those of bulk materials and single molecules [144,234,237-242]. Accordingly, these nanoparticles have drawn much attention as promising fluorescent labels for biology, so that the interaction of proteins and nucleic acids with the CdS and ZnS quantum dots has been intensively studied (see [242-248] for reviews). It is noteworthy that the ZnS particles obtained from hydrothermal vent plumes could pass through 200 nm filters [249] and, hence, can be reasonably categorized as nanoparticles.

#### Initial geological settings

An overall picture of primordial Earth, as based on available data, has been given in Section 2.1. The question that is crucial for the Zn world scenario is whether atmospheric pressure was in the range of 10–100 bar when life on Earth emerged (at this pressure range, hot hydrothermal fluids surging at sub-aerial settings would have been enriched in Zn; see above). It is generally accepted that life on Earth was unlikely to emerge before the end of the impact bombardment 3.9 Ga ago [25,26,80,93,95,96]. Since reliable biogenic fossils are dated to 3.4–3.5 Ga [135-139], life is believed to have emerged from 3.9 to 3.5 Ga [25,26,34,80,93,95,96]. Concerning the primordial atmospheric pressure, the following estimates have been put forward. Atmospheric pressure before condensation of the first ocean ca. 4.4 Ga ago has been estimated as ≥200 bar; this ocean condensation would have lowered the pressure to about 100 bar [94,100]. Accordingly, each evaporation of ocean that may have been caused by massive bombardment from 4.3 to 3.9 Ga would have transiently increased the atmospheric pressure. Atmospheric pressure 3.3 Ga ago has been estimated to have been in the range of 2–6 bar, depending on the assumed concentration of methane in the atmosphere [97]. It is remarkable that these estimates are compatible with the atmospheric pressure values in the range from ca. 100 bar to ca. 10 bar in the time window from 3.9 to 3.5 Ga. Hence, the time window when the hydrothermal ZnS could precipitate at sub-aerial settings overlaps with the time window when life is believed to have emerged on Earth.

The conditions under which the first cells emerged could be read from the chemical composition of cellular cytoplasm that apparently tends to maintain the ancient chemical milieu [25,34,84,85]. Archaeal, bacterial, and eukaryotic cells all show elevated concentrations of potassium, magnesium, phosphate, and certain transition metal ions (see Table 1). The elevated amount of transition metals, as already noted, could be attributed to the emergence of life in hydrothermal settings [25,93,113-115,177]. Not in contradiction with this attribution, the elevated amounts of K^+ ^and Mg^2+ ^might reflect the involvement of volcanic activities: elevated and positively correlated amounts of potassium and magnesium are typical of certain volcanic rocks [250].

The high phosphate concentration in cells might indicate the abundance of phosphorus compounds in the primordial waters [85,251,252]. Since the concentration of phosphate in sea water is low (the phosphates of Ca and Mg are poorly soluble in water), it has been argued that more reduced compounds such as hypophosphite (phosphinate, PO_2_^2-^) and/or phosphite (phosphonate, PO_3_^3-^), which have better solubility in sea water, could have been abundant in the primeval, more reduced ocean [252-258]. This suggestion is supported by findings of diverse systems of hypophosphite and phosphite oxidation in prokaryotes (see [259] for a review).

The high extent of hydrothermal Zn delivery to the surface of Earth during the earliest stages of its history is reflected in the association of present day major zinc ore deposits (built of ZnS) with Archean oceanic spreading centres and island arc terrains [260,261].

*In summary*, these features point to environments similar to those in which VMS deposits originated (see [187,262] for reviews). The largest VMS deposit is believed to be the Iberian Pyrite Belt, which is 200 km long and 40 km wide. The oldest VMS deposits discovered so far, dated 3.2–3.5 Ga, are at the Pilbara Craton of West Australia [25,263,264]. Remarkably, besides its huge zinc content (zinc lenses) [265,266], the Pilbara Craton also contains filamentous microfossils [267,268]. It is tempting to speculate that the Pilbara Craton could serve as a rough model of an environmental setting where life could have emerged earlier, at the end of the Hadean, when ZnS could precipitate, because of high atmospheric CO_2 _pressure, within reach of solar UV radiation.

#### Photosynthesis in the Zn world

The ZnS-mediated photosynthesis of diverse organic compounds from CO_2_, in view of its potential practical application, has been studied intensively, starting in the early 1980s [144-146,232,236,269-273]. Besides the reduction of CO_2 _to formate (see Fig. 1 for a scheme), the photosynthesis of dicarbonic, tricarbonic, and tetracarbonic acids has also been shown [146,155]. Yanagida and co-workers have reported the photoreduction of diverse acyclic and cyclic ketones to the corresponding alcohols [274,275] and the ZnS mediated formation of diethylamine from ethylamine [192]. It is noteworthy that the highest quantum yields of CO_2 _photoreduction, in the range of 10–80%, were obtained, by various research groups [144,145,148,149,155,273], with ZnS nanoparticles – analogues to those ejected by hydrothermal vents. These nanoparticles are particularly photosynthetically active since the probability of electron trapping at the surface increases with the surface/volume ratio [241,276]. Studies of the photoelectrochemical reduction of CO_2 _at *p*-type semiconductors showed that at high CO_2 _pressure the rate of reduction was principally limited only by light intensity [277].

While studying photochemical hydrogen production in suspensions of ZnS particles (with quantum yields reaching 90%), Reber and Meier found that the most stable, long-lasting hydrogen production was observed when a mixture of sodium sulfide and sodium hypophosphite was used as a hole scavenger [278]. This system appears to be very interesting in the evolutionary context. Here the accumulation of disulfide ions (S_2 _^2-^) that could be caused by sulfide (S^2-^) oxidation by photogenerated hole (h^+^) via a sulfide anion radical (S^**·-**^) as an intermediate according to the equations(1)(2)

was prevented by the reductive action of hypophosphite that yielded phosphite, so that sulfide was recycled:(3)

After all the hypophosphite wass consumed, hydrogen formation proceeded further, although slower, owing to the further oxidation of phosphite into phosphate [278]:(4)

The high efficiency of (hypo)phosphite as a hole scavenger was also demonstrated by Kanemoto and co-workers, who studied, in addition to the photogeneration of hydrogen, the photoreduction of CO_2 _by ZnS [273]. As already noted, hypophosphite and phosphite may have been abundant in the primordial ocean [252-254,256-258] and could participate in the primeval photosynthesis. Moreover, phosphate ions, ultimate products of the (photo)oxidation, owing to their affinity to metal sulfides [279], would have remained adsorbed at the ZnS surface, providing a specific phosphate-rich reaction milieu.

The aforementioned photo-oxidation of sulfur aniones can eventually lead to disruption of the ZnS crystals and release of Zn^2+ ^ions that could not be completely prevented even by efficient hole scavengers [271]. In the context of primordial photochemistry, such a photocorrosion would have led to the continuous rejuvenation of the ZnS surfaces and the formation of fresh (photo)catalytic interfaces. In addition, photocorrosion would have continuously released Zn^2+ ^ions, keeping their concentration at the illuminated ZnS surfaces high.

Besides reducing organic compounds and catalysing condensation reactions, semiconductors can drive the photoreduction of dinitrogen to ammonium. This reaction has been demonstrated with preparations of CdS [280] and TiO_2 _[281-284]. The photoreducing capacity of ZnS is higher than that of CdS and TiO_2 _[154,190], and therefore one would expect that primordial ZnS systems were capable of reducing dinitrogen to ammonium as well, thus complementing the ammonium content of hydrothermal fluids. The interaction of formate, produced upon CO_2 _reduction, with ammonium could yield formamide, which could serve as a universal building block for the (photocatalyzed) synthesis of both nucleobases and amino acids [285].

*Taken all together*, these data support the suggestion that porous ZnS edifices, when formed at the UV-irradiated surface of primordial Earth, could photoreduce CO_2 _and other chemical compounds. On a geological scale, the productivity of this photosynthesis may have been immense.

#### First settlers in the ZnS world

The suggested accumulation of complex but photostable π-systems in the UV-illuminated environments is in agreement with the presence of polycyclic aromatic hydrocarbons in meteorites [194,286], as well as with the reported synthesis of nucleotides from simpler precursors at the outer surfaces of Russian spacecraft [287]. Generally, one would expect that complex organic compounds would break into pieces in outer space. This is not the case; instead, the "pieces" join together at surfaces to build complex aromatic π-systems that apparently are more stable than simpler compounds against diverse types of cosmic radiation.

Senanayake and Idriss showed that TiO_2 _surfaces catalyzed a UV-powered transition of formamide into nucleobases [196]. These results document the ability of seemingly "destructive" UV light to drive syntheses of increasingly complex compounds at semiconducting surfaces.

Sutherland and co-workers have obtained activated pyrimidine ribonucleotides from cyanamide, cyanoacetylene, glycolaldehyde, glyceraldehyde, and inorganic phosphate in a reaction that bypassed free ribose. The synthesis yielded activated ribonucleotide β-ribocytidine-2',3'-cyclic phosphate as a major product and several co-products [17]. Prolonged irradiation of this mixture by 254 nm UV-light caused the destruction of various co-products and the partial conversion of β-ribo*cytidine*-2',3'-cyclic phosphate into β-ribo*uridine*-2',3'-cyclic phosphate. The authors concluded that there must be some (photo)protective mechanism functioning with the two natural nucleotides but not with other pyrimidine nucleosides and nucleotides [17].

The reason for the particularly high photostability of nucleobases has recently been clarified [164,165,288-293]. Two types of photochemical reaction paths, which lead to an extremely fast transition from the excited state into the ground state, have been identified, namely (i) the torsion of certain C-N bonds of rings and (ii) the de-protonation of azine or amino groups [164,292,293]. Both mechanisms require nitrogen atoms in the rings, which might explain the rather counter-intuitive (for a CO_2_-dominated environment) selection of nitrogenous compounds as constituents of RNA. More recently, it has been argued that the high photostability of nucleobases is not affected by their alkylation; such alkylated, mostly methylated, derivates (known as minor nucleobases) are found in the structures of tRNA and rRNA [294]. Together, major and minor nucleobases could represent the initial pool of primeval photostable compounds from which the major nucleobases were gradually selected by evolution [22].

To survive, the RNA-like oligomers should be resistant not only to solar UV light but also to the (photo)chemical activity of ZnS itself. The reducing potential of photoexcited ZnS is in the range of between -1.0 V and -2.0 V, depending on the crystal structure [149,154,295]. This is low enough to reduce CO_2 _(see Fig. 1), but insufficient to reduce any of the nucleobases that all have reducing potentials of less than -2.0 V [296,297]. On the other hand, as indicated by the energy diagram of Fig. 1, the holes that are formed in photoexcited ZnS have an oxidizing potential of > 2 V [154,190] and can potentially oxidize almost any adsorbed organic molecule, including nucleobases or nucleotides. Such an oxidation, however, is unlikely for two reasons. First, since ZnS is an *n*-type semiconductor, the holes, unlike electrons, are not mobile [298]. Second, ZnS contains intrinsic electron donors, namely sulfur anions (S^2-^), which should "outrun" external electron donors and reduce the immobile holes, yielding sulfur anion radicals, S^**·-**^[298,299]. The formed S^**·-**^radicals could either dismutate according to eq. 2, with the formation of disulfide anions [300], or they could be reduced by external hole scavengers (see eqs. 3 and 4 and [278]), or they could interact with organic compounds yielding their sulfo-derivates; in any case, however, they should not be able to oxidize nucleobases or nucleotides that all have oxidizing potentials above 1.2 V at neutral pH [301].

Photochemical damage from ZnS was also unlikely. Nucleobases absorb light at 260–270 nm and emit it at 300–310 nm [159,302]. ZnS nanoparticles absorb light in a broad range up to approx. 350 nm and emit light at 420–470 nm [144,303]. Therefore the radiation energy could be transferred from the adsorbed nucleotides to a ZnS template (and thus contribute to the CO_2 _reduction), but not in the reverse direction. Hence, the quanta directly absorbed by the ZnS templates would not damage the adsorbed RNA-like replicators.

Besides nucleobases, the primeval RNA-like polymers may have contained ribose and phosphate entities. Ribose may have been abundant as one of the products of the autocatalytic "formose" reaction, which was discovered by Butlerov in 1861 [304] and which yields a mixture of pentoses and hexoses from formaldehyde. Although Butlerov's reaction remains the only known autocatalytic reaction that does not require specific catalysts, the importance of this reaction for prebiological syntheses has been questioned since the yield of ribose in the product mixture is usually low. Recent studies have shown, however, that the yield of ribose can be selectively enhanced by the presence of phosphate in the reaction medium [305], by UV illumination [16], and by conducting the reaction in the presence of catalytic mineral templates [306]. More recently, it has been demonstrated that the yields of pentoses increase to 60% and those of the ribose proper rise to 20% in the presence of a zinc-proline complex as a catalyst [15]. The Zn world settings may have favoured autocatalytic ribose formation from photosynthesized substrates by providing mineral templates, UV irradiation, and plenty of Zn^2+ ^ions as catalysts.

It has been argued that biological stereoselectivity (homochirality), i.e. the utilization of only particular optical isomers by living organisms, could have begun from the selection of a particular D-isomer of ribose – since nucleobases and phosphate groups are non-chiral [307]. Generally, homochirality cannot be completely explained in the framework of the "primordial soup" concept [3-5], because stereoisomers are chemically indistinguishable in a homogenous solution. At a surface, however, the properties of two stereoisomers could differ [307], as first pointed out by Goldschmidt, who has suggested that mineral surfaces were involved as templates in abiogenesis [35,308]. The two other tentative mechanisms of primordial stereoselectivity are (i) photoselection by polarized UV light (see [309] and references therein), and (ii) enantiomeric autocatalysis (see [310,311] for reviews). An example of this latter mechanism is the Soai reaction, where heteroaromatic aldehydes react with organo-zinc compounds, yielding respective alcohols, which in turn serve as asymmetric catalysts for their own formation. If one of the substrate enantiomers is present even in small excess, the autocatalytic reaction can yield the corresponding product with up to 95% enantiomeric excess [312-314]. Although the mechanism of the Soai reaction remains unclear, Zn^2+ ^ions might be important – zinc-proline complexes were also shown to mediate stereoselective catalysis of aldole reactions in water [315]. At this point it is appropriate to mention that the Zn world settings could support all these mechanisms of stereoselectivity by providing (i) electrically charged surfaces with regular patterns of positive and negative charges, (ii) Zn^2+ ^ions that could build potentially catalytic complexes with diverse organic compounds, and (iii) UV light that would become polarized after passing through the ZnS crystals [316]. These factors resemble strikingly the aforementioned features that increased the ribose yield in the autocatalytic Butlerov's reaction. Although the relative importance of the above-named features for prebiological stereoselective and/or autocatalytic syntheses of D-ribose and other sugars remains unclear, they can be experimentally tested: the stereoselective (photo)catalysis of diverse organic reactions at the surface of ZnS is an established approach in photochemistry [191,317-320].

Turning to the suggestion that the primordial waters were enriched not in phosphate but in the better soluble phosphite, it is worth noting that phosphate and/or phosphite groups may have catalyzed prebiotic syntheses [17,32,305,321,322], serve as bridges upon connecting nucleobases with metal sulfides [279,323], participate in UV-driven photochemical reactions [324], prevent hydrolysis of the first oligomers [325], and interact with diverse organic molecules, yielding their phosphorylated derivates [252,326]. It is tempting to speculate that the oxidation of phosphite ions, as hole scavengers, upon ZnS-mediated photosynthesis (see above) may have been coupled with their involvement in polymerization reactions as catalysts, yielding surface-confined oligomers connected by phosphate groups [252,255,327].

The Zn world concept is consistent with a direct assembly of polynucleotides at ZnS surfaces either from stacked nucleobases, ribose molecules, and phosphate/phosphite linkers or, perhaps, even from simpler parts (as exemplified by Sutherland and co-workers [17]). It is plausible that diverse (photo)synthetic pathways may have been realized at the ZnS surfaces with the output being essentially determined by photoselection of most stable compounds.

The light-induced energy transfer between adsorbed organic dyes, single nucleotides, and polynucleotides on the one hand, and ZnS/CdS templates on the other hand, has been intensively studied [242-244,247,328]. In one case at least, it has been quantified that an organic dye *fac *tris(2-phenylpyridine) iridium, when adsorbed on a ZnS surface, can serve as an antenna and increase the amount of quanta captured by ZnS [329]. This result supports the possibility of a positive feedback between the number of UV-light-absorbing zymes in a ZnS compartment and the yield of photosynthetically produced metabolites.

DNA and RNA are capable of long-range energy transfer along stacked nucleobases, over tens of nucleotide pairs [330-333]. In the context of the primordial Zn world, this property could have been useful: if a UV quantum hit a nucleotide having no direct contact with an energy-absorbing template, then, owing to the coupling between the stacked and paired nucleobases, the excess energy would still have promptly sunk into the ZnS substratum.

The suggestion that RNA-like polymers could bind, via negatively charged phosphate groups, to ZnS surfaces is supported by the ready adsorption of nucleic acids on the ZnS/CdS nanoparticles [243,247,328,334-340] The assembly of RNA-like polymers at the ZnS surface should be greatly facilitated by the complementary match between the patterns of electrically charged groups at the surfaces of polynucleotides and ZnS, respectively. Such a matching was shown for the CdS nanoparticles, which formed spontaneously from added cadmium salts in the presence of polynucleotides as templates [341,342]. This match may have a straightforward explanation: the distance between the positively charged Cd^2+^/Zn^2+ ^ions of 0.58/0.54 nm, respectively, in the nanoparticles [343,344] is similar to the distance of 0.58–0.59 nm between the phosphate groups in the RNA backbone [345].

Wächtershäuser has suggested that a regular mesh of electric charges at the surface may have made the primeval polymerization thermodynamically more favourable by attracting reactants and arranging them appropriately relative to each other [124]. De Duve and Miller have countered him by noting that if the polymerization at the surface was thermodynamically more favourable than in the bulk solution then the polymers could not detach without "paying" the respective energy fee and would have remained confined to the surface [200]. As argued above, such confinement would have prevented photodestruction of the polymer molecules and favoured their interactions at the ZnS surfaces. It is worth noting that a mechanism of thermodynamic confinement is actually exploited by nature upon the synthesis of ATP from ADP and inorganic phosphate by membrane ATP synthases. While the free energy of ATP synthesis in water is about +50 kJ/mol, molecules of ATP build up spontaneously in the enzyme active site, the reaction being facilitated by a positively charged arginine residue [346,347]. The molecules of ATP, however, remain tightly bound: they can leave the catalytic pocket only after the free energy of membrane potential is used to open the pocket and to reorient the arginine residue; ATP can then dissociate into the water phase [346]. The phosphate group in ATP is linked by a phosphoester bond that is similar to those connecting the nucleotides in RNA and DNA. Hence, as compared to a reaction in a bulk-water phase, the formation of phosphoester bonds can indeed be favoured when the reactants are appropriately arranged and a positive charge is present nearby.

Orgel and co-workers have studied the polymerization of guanosine 5'-phosphorimidazolide on a polycytidylic acid template in the presence of a variety of metal salts [198,348]. They found that "*none of the metal ions investigated behaved like Zn*^2+ ^*in favoring the formation of the naturally occurring 3'-5' linkages*" (quoted from [198]). A specific role of Zn^2+ ^ions in shaping the proper 3'-5' linkages follows also from the recent work of Hadley and co-workers. They selected deoxyribozymes that could ligate RNA and found that the native 3'-5' linkages were built only by those deoxyribozymes that were dependent on zinc [349]. In addition, mostly 3'-5' bonding has been observed upon the radiation-driven polymerization of nucleotides at the surface of volcanic rocks [350]. Hence, both Zn^2+ ^ions and mineral templates seem to favour the formation of proper 3'-5' bonds.

The emergence of longer RNA strings could have proceeded not only via polymerization but also through spontaneous rearrangements of RNA sequences that may progress in the absence of any enzymes or ribozymes [351-353]; such rearrangements may have dramatically accelerated evolution [57,354].

*In sum*, the (photo)catalytic properties of Zn^2+ ^ions and Zn-containing substances could have shaped the first life forms. While the photochemistry of ZnS crystals could have governed the nature of photosynthesized compounds and that of their photo-derivates, the catalytic properties of Zn^2+ ^ions may have determined the particular traits of the first (bio)molecules, such as the choice of 3'-5' linkages for RNA polymers.

#### The first replicators and the emergence of proteins and enzymes

Earlier, while hypothesizing on the selective advantage of paired RNA strands over those unpaired in primordial UV-illuminated settings [112], we built on empirical evidence of the higher UV stability of double-stranded RNA samples as compared to single-stranded ones [204-206]. Recently, the physical background of this higher photostability has been clarified. For the nucleotides that form a Watson-Crick pair, the lifetime of the excited state has been estimated to be as low as a few femtoseconds [355,356], i.e. ca. one hundred times shorter than that of single bases [159-163]. This extremely short lifetime has been attributed to excited-state deactivation via electron-driven proton shuttling between the bases [357-359]. It is noteworthy that other possible (not Watson-Crick) conformers of paired nucleobases have not shown these unique photochemical properties [355]. It has been suggested "*that the biologically relevant Watson-Crick structures of GC and AT are distinguished by uniquely efficient excited-state deactivation mechanisms which maximize their photostability*" (quoted from [164]).

It is necessary to emphasize that neither the high photostability of single nucleobases [159-165,288-293], nor the even higher stability of their Watson-Crick pairs [164,355,356], nor the aptitude for long-range energy transfer along stacked nucleotides [330,333,360] have anything to do with the current functioning of RNA and DNA in the transfer of genetic information. All these traits, however, support the suggested involvement of UV light as a selecting factor during the initial stages of evolution [105,112,133,159,164]. This selection pressure would have favoured the relative enrichment of photostable aggregates built of paired RNA-like strands with a potential for self-replication.

The idea of primeval RNA-based self-replicating aggregates got additional support from the work of Lincoln and Joyce who presented a system of two ribozymes that catalyzed synthesis of each other from a total of four oligonucleotide substrates. These cross-replicating RNA enzymes underwent self-sustaining exponential amplification in the absence of proteins or other biological materials [72].

The data on specific affinity of various proteins to the ZnS/CdS nanoparticles [243,246,361-364] suggest that ZnS surfaces could serve as templates for the synthesis of the first polypeptides or as baseplates for the first protein synthases. A primeval RNA machine capable of making peptide bonds has been recently unveiled by Bokov and Steinberg based on a detailed analysis of the structure of the modern ribosome [73]. As the first protein synthases most likely began by generating random polypeptides [73], it might be useful to consider – in a search for tentative sequence-independent functions of the first proteins – the traits that are common for all polypeptides. To perform sequence-independent functions, the first polypeptides should have been able to interact with polynucleotides via their backbone atoms. Carter and Kraut have suggested that a protein chain can fit into the minor groove of an RNA helix, with hydrogen bonds being formed between the ribose 2'-hydroxyls and carbonyl oxygen atoms of peptide bonds, in an interaction that is RNA-specific and is not possible in the case of DNA [365]. Indeed, hydrogen bonding between the protein backbone oxygen atoms and the ribose 2'-hydroxyls has been found in many RNA-protein complexes [366]. This ability of proteins to block the 2'-hydroxyls of ribose entities may have increased the life span of primordial RNA polymers. The aforementioned ability of Zn^2+ ^ions to catalyze the formation of proper 3'-5' linkages [198,349] implies the ability of Zn^2+ ^ions also to catalyze cleavage of these linkages. Butzow and Eichhorn have noted that the Zn^2+^-catalyzed cleavage of RNA starts from the binding of a Zn^2+ ^ion between the 2'-hydroxyl group of ribose and the phosphate group [367]. It is tempting to speculate that the first polypeptides could protect the RNA molecules from metal-catalyzed cleavage by preventing the binding of Zn^2+ ^ions to the 2'-OH groups of ribose.

The (photo)stability of polynucleotides and proteins, as argued by Sobolewski and Domcke [164], is ensured by reversible proton relocation within picoseconds between the Watson-Crick-paired nucleotides and along the protein hydrogen bonds, respectively. In other words, the reshuffling of protons along hydrogen bonds can promptly split a large "captured" energy quantum into many small, non-hazardous heat quanta, both in nucleic acid polymers and in proteins [164]. In a UV-irradiated environment, this feature should favour the selection of structures with many intrinsic hydrogen bonds, such as paired RNA or DNA strands, protein α-helices and β-sheets, as well as hydrogen-bonded RNA-protein aggregates. Turning to the previously discussed relation between the stability of polymers and their ability to perform work, the shuttling of protons along hydrogen bonds might be considered as a kind of *work *that could be carried out at picoseconds, i.e. much faster than any bond dissociation could take place. It is noteworthy that the acid-base catalysis, seemingly prevalent in ribozymes and enzymes [368-371], consists of proton relocation(s) between the donor and acceptor groups [372-374]. Accordingly, the selection of hydrogen-bonded systems as more (photo)stable ones could have paved the way to the first catalytic centres, first in ribozymes and then in proteins.

Several authors, based on quite different premises, have argued that the first genetically coded amino acids were the neutral and acidic, starting from glycine, alanine, valine, and aspartate, with the positively charged amino acids being added later [46,47,53,375,376]. In the absence of positively charged amino acids, metal ions and, in particular, Zn^2+ ^ions, could have been recruited by the first enzymes as catalytic Lewis acids (Manfred Eigen, personal communication).

*In summary*, the illuminated ZnS settings may have contributed to the emergence of first replicators and enzymes by favouring the formation of photostable, hydrogen-bonded structures, by serving as templates upon syntheses, and by providing Zn^2+ ^ions as potent catalytic cofactors.

#### Colonization waves

Chetverin and co-workers have introduced and studied RNA colonies that grew and propagated on gels or other solid media provided that RNA replicases and ribonucleoside triphosphates were present [351,377,378]. It has been explicitly noted that such experimental systems might, in fact, model the amplification and propagation of the first replicators in primordial environmental settings [57,351,354]. Further indications of primeval RNA life might be the participation of tRNA molecules as catalysts in several metabolic reactions (see [23] and references therein) and interactions of RNA molecules with metabolites [379,380], in particular in the case of riboswitches [381,382].

As argued by Koonin and co-workers, viral hallmark genes shared by many groups of RNA and DNA viruses – but missing in cellular life forms – might be relics from the pre-cellular RNA/protein world [61]. Moreover, the specific affinity of many metabolic enzymes to RNA [383,384] could also stem from the life forms that were built of RNA and proteins.

Last but not least, porous, inhabited ZnS settings still persist around deep-sea hydrothermal vents; their dwellers, mostly archaea, have been characterized on several occasions [179,181].

*At the end of this chapter*, it is fair to note that a large part of the cited evidence comes from nanotechnology research in which ZnS/CdS nanoparticles are paradigmatic objects of study. Still, while sifting through the literature on the interaction of nucleobases, polynucleotides, and proteins with ZnS/CdS nanoparticles (see e.g. [242-244,246,247,328,334-342,361-364,385]), it is difficult to avoid the impression that the "*intrinsic affinity of (poly)nucleotides for semiconductor surfaces*" (quoted from ref. [334]) is particularly specific. In the framework of the Zn world scenario, it is tempting to speculate that, while interacting keenly with the ZnS/CdS surfaces, the biological polymers might recall their evolutionary past.

### Evolutionary continuity in the Zn world

A scenario for the evolution of a complex system must consist of plausible elementary steps, each conferring a distinct advantage (Darwinian continuity principle; see also ref. [27,67,218]). Furthermore, these steps also have to be physically plausible, which implies, in addition to the correspondence with physical laws, the continuity of underlying mechanisms and driving forces. Below, the interplay between the Darwinian and physical continuities in the Zn world is considered in more detail.

#### Multifarious energetics of the Zn world

Admittedly, the least physically plausible step in the available origin of life scenarios is the abiogenic emergence of complex polymers capable of replication and catalysis. As discussed above, at least one type of polymer should have been continuously emerging under primeval settings to enable a selection of first catalytic entities that could gradually develop an ability to synthesize other types of polymers. Based on the aforementioned arguments, these primary polymers could be related to modern RNA. The synthesis of RNA and DNA molecules in modern organisms is driven by the hydrolysis of ATP molecules and carried out by sophisticated enzyme systems. Turning to the primordial Earth, one has a typical "chicken and egg" paradox: on the one hand, high-yield polymerization would seemingly have required specific machinery for coupling with exergonic reactions, yet, on the other hand, this machinery was likely to have been absent before the first catalytic polymers have emerged. One of the virtues of the Zn world scenario is the possibility to funnel UV energy into polymerization reactions. In addition, the thermodynamically very favourable oxidation of phosphite to phosphate could potentially provide free energy. Furthermore, the polymerization at ZnS surfaces could be more favourable than in the bulk-water phase (see above). Nevertheless, it is unclear whether these factors alone could have been sufficient for maintaining a notable steady-state population of RNA-like polymers – needed as starting material for further evolutionary transformations. The results of our earlier Monte-Carlo simulations of primordial photochemistry [112], suggest one more, rather paradoxical, way to channel external energy into the synthesis of increasingly complex compounds.

The aim of the simulations was to quantify the significance of the UV protection mechanism for the evolution of primordial RNA-like polymers by computer modelling of the polymerization of sugar-phosphate monomers in the presence of nucleobases.

1) "Dark" simulations (with "virtual" UV light switched off and a polymerization constant of > 1) yielded polymers that, as expected, did not carry nucleobases, since the equilibrium constant of their attachment to the sugar-phosphate units was taken, in accordance with the actual situation, as <<1 (see [112] for further details). Despite a high polymerization constant, the elongation of polymers was not unconstrained. The explanation as suggested by D. Cherepanov (personal communication) is the following: if a linear polymer contains *n *monomers, the binding of new monomers can proceed only at the two terminal positions, while dissociation can implicate any bond, i.e. it can proceed at (*n*-1) positions. Hence, the probability of dissociation should increase with the value of *n *and prevent the formation of excessively long polymers. This straightforward consideration, as well as the simulation results, discount the common belief that the longer polymers could have built up solely by means of their postulated higher stability against hydrolysis as compared to shorter polymers (see e.g. [386]).

2) After turning the virtual UV light on and in the absence of UV protection, with UV quanta being able to cleave the sugar-phosphate bonds, the polymerization yield dropped severely, and the extent of nucleobase incorporation in the polymers remained close to zero.

3) With the UV protection "switched on" (so that the binding of a nucleobase to a sugar-phosphate unit decreased the probability of UV breakage by a factor of 30), the length of formed polymers increased dramatically. Even more importantly, under these conditions the fraction of nucleobase-carrying sugar-phosphates increased significantly (up to ~0.5; see [112]).

4) We also simulated the effect of the partial funnelling of UV energy into the condensation reactions by assuming that with a probability as small as 9 × 10^-8 ^a photogenerated radical could bind a nucleobase in a proper, productive way. The resulting boost for the formation of oligonucleotides was remarkable concerning the length of the formed polymer chains and the extent of nucleobase incorporation into the oligomers, so that the long polymers were built predominantly from nucleobase-carrying sugar-phosphate units (i.e. nucleotides; see [112]).

Result no. 4 is physically trivial because it follows from the enabled channelling of the virtual solar energy into the synthetic reactions. However, the increase in the relative fraction of longer, nucleobase-carrying polymers solely in response to switching on the UV protection (result no. 3) is not trivial at all, because in this case the utilization of radiation energy for synthesis was not "permitted". Here, the increase in the fraction of complex polymers was due to the breakage of the less photostable molecules by the virtual UV light. Since the number of molecules in the Monte-Carlo simulation was limited, the relative fraction of more photostable and, accordingly, more complex polymers increased. Therefore – and this is the key point – the enrichment in more complex RNA-like polymers was driven by the photo-dissociation of their less stable counterparts, in the absence of any direct coupling between the energy flow and the synthetic reactions.

From the experimental viewpoint, the results of this modelling might be related to the aforementioned finding of Sutherland and co-workers that prolonged UV illumination of the reaction medium containing a set of pyrimidine nucleotides and nucleosides led to the enrichment of photostable natural nucleotides on account of non-natural compounds [17]. From the theoretical viewpoint, the described mechanism of "indirect" energy utilization might be related to the so-called Landauer principle. Rolf Landauer, while working for IBM, demonstrated that, contrary to common belief, information *per se *could be created in a reversible way, i.e. "for free". Further analysis has shown, however, that energy would then be required to erase all the intermediate steps of such a reversible computation from the computer memory [387-390]. Accordingly, the creation of information requires energy in any case, but the energy can be utilized in two different ways: (i) for creating the information itself and/or (ii) for erasing the "garbage" from the system. It is noteworthy that the need for "erasing energy" arose because Landauer had considered a physically realistic computer with a limited number of memory units. At least on this point, Landauer's formalism corresponds to our Monte-Carlo simulation that also invoked a limited number of building blocks [112].

Several authors have argued recently that the physical model of Landauer might be related to biology, in particular to creating and maintaining increasingly complex life systems [92,391-393]. As noted by Danchin, "*creation of information requires many steps, it starts from a given complex of dynamic interacting entities, that progressively transforms into variants, among which some have a higher information than that of the original complex*" [92]. It would seem that this quotation is equally applicable to computation processes and to the evolution of first RNA-like polymers on primordial Earth. Then, however, the emergence of increasingly complex RNA-like polymers could be driven by the energy-consuming, selective erasing of less "perfect" variants. Danchin writes that this type of selection process has to "*actively discriminate between entities that are in some degree functional and those that cannot function. This is because the process needs to avoid destroying the elements that carry increased information, and this is where energy comes in. Energy has to be consumed to make innovations stand out, in a discriminant process: energy is used to prevent degradation of functional entities, permitting destruction of the non-functional ones*" (quoted from [92]). It is noteworthy that Danchin has invoked Landauer's formalism to describe the aging phenomena. Still, this approach seems to be applicable to the primordial RNA-like polymers in the Zn world, provided that we consider the more photostable constructs as "functional" ones and the solar UV radiation as the selecting and erasing energy input. In fact, since the more complex, longer polymers have more opportunities to fold or aggregate with the formation of UV-stable double strands, the increase in photostability should be coupled with the increase in complexity and, accordingly, in the information content (see also below). Incidentally, the interplay between supporting and erasing energy fluxes, which is apparent in the case of photostable RNA-like molecules, might represent the physical essence of biological evolution (see [92,394,395] for related discussions).

In any case, the competition between the RNA-like oligomers should have been tough in the primeval Zn world. The ZnS compartments (pores) could accommodate only a limited number of polymers, so that the enrichment of more photostable polymers – driven by the photo-dissociation of the less fit ones – must have been pronounced. In addition, the survival of a molecule depended not only on possession of apt UV quenchers but also on binding to a mineral template capable of absorbing excess radiation energy, so that primordial polymers would have competed for free surface patches. In this competition, the RNA-like polymers that, as argued above, could match the ZnS surfaces electrostatically would have gained an advantage.

It is possible to conclude that in the Zn world the energy of solar UV radiation may have been utilized in three different, although complementary, ways, namely: (i) for a high-yield, ZnS-mediated photosynthesis of diverse organic compounds, (ii) for fortuitous photopolymerization events, and (iii) for relative enrichment of the photostable, surface-adsorbed RNA-like polymers via the systematic photo-dissociation of more labile molecules.

#### Reproduction and replication in the Zn world

A few authors have noted that the origin of life could be a two-step process, with the emergence of the first replicators being facilitated by the pre-existence of out-of-equilibrium settings favouring their formation (see e.g. [27,396,397] and references therein). Dyson has hypothesized that the emergence of the first life forms capable of error-free *replication *may have been preceded by simpler *reproducing *systems where interacting chemical cycles, supported by external energy sources, could reach homeostasis and persist over time. He has even speculated that such reproducing systems could have evolved and attained complexity comparable to that of modern cells [398]. The Zn world settings can be considered out-of-equilibrium *and *reproducing, with their persistence being guaranteed by the hydrothermal activity of Earth and by solar energy. The precipitation of ZnS around hot springs would have led to a continuous *reproduction *of new, empty compartments [61,176,219] where the selection of replicators could proceed on a geological timescale. In such settings, photostable compounds could have continuously emerged and undergone photoselection for millions of years, until their particular mutual interaction yielded an aggregate capable of robust self-replication. The emergence of life can therefore be considered not as an accident, but as a natural outcome of the interplay between solar radiation and particular geothermal processes on primordial Earth.

The role of reproducing, illuminated ZnS settings may have been in enabling the (photo)selection of the first replicating entities. Hence, this solar-powered selection may have been a link between abiogenic reproducing systems and the first replicators.

#### The origin of meaningful complexity in the Zn world

Since the organisms, and not the polynucleotide molecules, are the subjects of evolution, some authors have noted that nucleotide strings (e.g. the genes that code particular proteins) are meaningless unless the machinery that translates the DNA/RNA-coded information into the final products is explicitly taken into account, see [399-401]. The Zn world scenario suggests how the biological meaning could emerge gradually, being driven by (photo)selection, with the more photostable systems gaining selective advantage upon each step. Indeed, if we could trace, in a UV-irradiated environment, the fate of two RNA-like polymers of the same length and the same information content (see [402,403] for surveys of relevant definitions), we may find that the polymer that could fold into a structure with more double-stranded segments and/or to bind tightly to an energy-absorbing template could endure more hits by UV quanta. Therefore each polymer could be characterized by its (i) intrinsic complexity related to the probability of its emergence [402,403] and (ii) extrinsic complexity, the value of which would reflect its survival chances in given settings and could change depending on conditions. The more stable constructs, with higher complexity, would have had longer lifetimes and be steadily present in relatively larger numbers. Hence, they would have had more chances to interact with other stable constructs to form even more elaborate aggregates. At the moment when one such aggregate begins to replicate itself, the complexity of its constituents, thus far related to their survival chances in a UV-irradiated environment, becomes biologically meaningful. At the very same moment, the nucleobases – which were hitherto acting solely as efficient UV-protecting units – become the letters of the genetic alphabet. In this framework, the "meaning", in the biological sense, is intrinsically coupled with the survival chances under given circumstances and thus can precede the emergence of the translation machinery (that could have gradually developed later; see e.g. [46,47,58,67,73] for tentative scenarios).

Since polynucleotides do not last long in nature, replication can be considered as a way to preserve (accumulate) information before an information carrier – a polynucleotide strand – eventually breaks down because of thermal fluctuation or (photo)cleavage. It is likely that the first replicating systems invoked linear polymers because the replication of branched polymers – as a logical alternative – may have been mechanistically impracticable.

In summary, we have an example of a Darwinian function co-option [404]: the "irreducible complexity" of the first replicators can still be reduced by suggesting that their simpler "ancestors" underwent selection for a different, less structurally demanding aptitude (namely for their ability to survive in a UV-irradiated environment).

#### Physical continuity of life

Living organisms are routinely considered as systems that sustain themselves by converting "few" high-quality energy quanta into "many" low-quality, thermal quanta, this process being accompanied by entropy production, see [87,90,209,405]. These high-quality (or high-energy) quanta are those that contain portions of energy large enough to drive chemical reactions, such as the quanta of light in the UV and visible ranges or the energy portions that are released upon redox reactions. In such a framework, biological evolution could be understood as a way to optimize the utilization of high-energy quanta [90,209].

The evolution of energy conversion at the earliest evolutionary stages, e.g. in the RNA world, as well as the driving force(s) beyond this evolution have remained, however, undefined. In modern organisms, the conversion and storage both of the energy of light and of chemical energy are carried out by membrane enzymes that catalyze sophisticated chains of redox reactions (see e.g. [176,406-408] for reviews). It is noteworthy that (known) natural ribozymes cannot store either the energy of light or the redox energy [68]. Not surprisingly, the traditional concept of the RNA world has circumvented the question of exploiting the external energy fluxes.

Before addressing this question, it is useful to realize that the ability of high-energy quanta to drive chemical reactions does not require these reactions to be useful. For a living organism, the outcome from capturing a UV quantum may have been hazardous rather than beneficial. In the Zn world, where surface-confined photosynthesized molecules could not escape solar UV light, the first RNA-like polymers may have initially been selected not for their ability to exploit the high-energy quanta but for the ability to discard them promptly or, in other words, to "process" them without undergoing photodamage. The ability to discard the energy quanta is less structurally demanding than the ability to exploit them, so, as argued above, this ability may already have been inherent in simple organic molecules. It is tempting to suggest that the common energetic denominator of living organisms – throughout the whole evolutionary time span – may have been, not the ability to exploit high-energy quanta, but the ability to "deal" with them – in a general sense.

This suggestion makes it possible to follow a continuous evolutionary thread from the origin of life event to the modern struggle of mankind for energy. At the beginning of this thread, the first RNA-like polymers could have emerged as systems capable of discarding potentially hazardous high-energy quanta without undergoing damage. The energy requirements of the first replicators could have been covered by the breakdown of "nutrients" produced in photosynthetic but abiogenic reactions. The first heterotrophic organisms, while retaining the ability to discard excess energy, could have gradually developed abilities to control the high-energy quanta and to use them. It is plausible that solar energy, besides serving as a selective factor, may have occasionally promoted chemical reactions (e.g. as happens in natural photolyases [409,410] or in an artificial deoxyribozyme with photolyase activity [411]). The funnelling of high-energy quanta into useful reactions may have been profitable *per se *and, in addition, could have prevented undesirable overheating and denaturation. However, only the emergence of biogenic dielectrics, namely proteins and phospholipid membranes, enabled the storage of the energy of light in the form of charge-separated states, physically analogous to those involved in ZnS-mediated photosynthesis (see Fig. 1 and the accompanying article [226]). This development may have paved the way to the first phototrophic organisms in which membrane-embedded, energy-converting systems could have developed, supposedly, from the UV-protecting membrane proteins of the first cells [226,412]. It is noteworthy that modern chlorophyll-carying proteins, besides being involved in the storing and utilization of solar energy, retain the ability to protect cell DNA from the residual UV component of solar radiation [413], thus resembling the nucleobases that, in addition to serving as the letters of genetic code, continue to protect DNA and RNA backbones from UV damage [159]. In this framework, the gradually attained ability to exploit high-energy quanta of light can be considered as an evolutionary younger, as compared to their deactivation, and more sophisticated way of dealing with them. As the accessible energy assets ran out, living organisms had to "acquire new powers" (quotation from [1]), i.e. increase their complexity, if they wanted to access and exploit new energy sources (see [21,90,209] for detailed considerations of this point).

*In summary*, the emergence of life on Earth may be traced to the successful attempt of particular (hetero)cyclic molecules to overcome, in a joint effort, the hazards of solar UV rays that they could not escape.

### Relationship to other hypotheses on the origin of life

The suggested scenario invokes elements from different, even seemingly contradicting, hypotheses on the origin of life. This should not be surprising, since each of these hypotheses builds upon empirical data and/or certain theoretical bases. Accordingly, these empirical observations and theoretical considerations have to be accounted for by any scenario that claims to serve as a platform for further discussions in the field.

Oparin's hypothesis of a heterotrophic origin of life implied that the initially established metabolism could have resembled fermentation [3,4]. The rationale behind this supposition (see [26,28,29,34] for further development of this view) was straightforward: fermentation is a simple mode of metabolism that is inherent in the most primitive anaerobic organisms. We now know that catalytic chemistry beyond the breakage or formation of covalent bonds is indeed the least structurally demanding; in most cases only acid-base catalysis is involved, often enhanced by metal atoms [368-371]. Not surprisingly, reactions of this type can be catalyzed even by ribozymes, unlike many other chemical transformations that can be performed only by proteins. The Zn world scenario builds on this rationale and suggests that primordial metabolism, as catalyzed by the first ribozymes and enzymes, constituted a set of chemically simple transformations where reactions of bond breakage were coupled with reactions of bond formation, perhaps being mediated by group transfer events. Oparin, however, did not treat primordial energetics explicitly [3,4]. The formation of the first polymers was suggested to proceed spontaneously, without coupling to energy flow. Without such coupling, however, an unsupported formation of long polymers would contradict the laws of thermodynamics, as routinely noted by critics of Oparin's idea (see e.g. [83]). In contrast, the Zn world scenario invokes solar UV light as an energy source both for prebiological syntheses and for (photo)selection.

Haldane, in addition to a fermentation-like metabolism, which he postulated independently of Oparin, suggested that "*the first living ... things were probably large molecules synthesized under the influence of Sun radiation*" (quoted from [5]). Hence, on this point Oparin's and Haldane's hypotheses differ. In addition, Haldane argued that the complexity of the first organisms should be compatible to that of bacteriophages, thus anticipating some aforementioned modern views related to the RNA world concept [61,62,219]. On all these points, there is an agreement between the Zn world concept and Haldane's hypothesis. Nonetheless, Haldane suggested that life emerged in the homogenous phase (it was he who mentioned the "*hot, dilute soup*"). In contrast, the Zn world concept considers primeval life as a surface-confined phenomenon.

The Zn world concept is in accord with the RNA world (RNA life) hypothesis [37-73]; the RNA-based life forms may have inhabited the photosynthesizing, porous ZnS edifices that could help to (photo)select first RNA organisms, provide a shelter and nourish them. RNA organisms most likely inhabited the Zn world only in the beginning, being followed by RNA/protein life forms. Concerning RNA research, the involvement of ZnS surfaces suggested here implies that, upon modelling or simulating primeval events, electrically charged ZnS surfaces may be explicitly invoked as supporting (photo)catalytic templates (see also [414]). With their assistance, replication could have been carried out by relatively simple aggregates of RNA-like polymers. In addition, the first systems of protein synthesis, owing to supporting ZnS templates, may have been fundamentally simpler than their modern counterparts (see also [73]). The gradual decoupling from the ZnS surfaces and the emergence of enclosed organisms would have required more complex devices, such as primeval ribosomes, which, however, had time to evolve gradually from more simple predecessors – by recruiting new parts to functionally replace the ZnS baseplates.

The Zn world scenario incorporates elements from some "metabolism first" concepts, in particular from the "Pyrite world/Pioneer organism" concept of Wächtershäuser, who put forward a detailed concept of a two-dimensional primordial life on iron disulfide (FeS_2_, pyrite) surfaces at the sea floor [124-130]. The Zn world scenario exploits the suggestion of Wächtershäuser that the mineral surfaces may have promoted the abiogenic syntheses of the first polymeric molecules. In addition, the Zn world model builds upon the idea of Russell and co-workers on porous chimneys of the deep-sea hydrothermal vents as incubators for the first life forms [115-123]. In contrast with these two concepts, the Zn world scenario considers zinc sulphide – instead of iron sulfides, which can generate potentially harmful hydroxyl radicals [223,224] – as the "mineral of life", and locates the first life settings at the illuminated surface of Earth. Moreover, the suggested metabolism in the Zn world, which combines abiogenic photosynthesis with the primitive heterotrophy of the first replicators, is fundamentally different from the (bio)chemical mechanisms that were postulated for the two "iron-sulfide" worlds and based upon iron-catalyzed redox reactions (see the accompanying article [226] for more details on this point).

Kuhn and Waser have noted that the pores of primordial rocks could have served as compartments upon the evolution of the first RNA molecules [45]. A related attempt to merge the FeS compartments with the RNA life has recently been performed by Martin and co-workers [117,219], who have suggested that "*within a hydrothermally formed system of contiguous iron-sulfide (FeS) compartments, populations of virus-like RNA molecules, which eventually encoded one or a few proteins each, became the agents of both variation and selection*" (quoted from [219]). Experimental evidence of polynucleotide accumulation in simulated pore systems has been provided by Braun and co-workers [415]. The idea of porous natural settings as incubators for the first RNA-like life forms is used in the Zn world scenario. It, however, suggests that the function of "honeycombed" ZnS settings was not limited to compartmentalization but also included photosynthesis of metabolites, (photo)selection of most fit polymers, and (photo)catalysis of diverse reactions. In addition, the Zn concept implies that colonization of these settings by first life forms proceeded from the illuminated, photosynthesizing top to the bottom, in contrast to the scheme of Martin and co-workers in which life propagated from the bottom, "secure" part of the deep-sea hydrothermal FeS vent to the surface.

A few authors [19,416,417] have stressed the potential importance of high radioactivity on primordial Earth for the origin of life, albeit without suggesting how the energy of ionizing radiation could be used for biochemically relevant syntheses. The Zn world scenario incorporates such a mechanism: ZnS crystals can trap and transiently store, in a form of charge-separated states, diverse kinds of radiation energy, namely X-rays, electrons (as in displays), α-particles (ZnS was introduced as the first inorganic scintillator by Sir William Crookes in 1903), and so on. The excited electron(s) could then drive the CO_2 _reduction at the surface of ZnS crystals. Hence, ZnS crystals show a unique ability to convert diverse kinds of energy into the chemical energy of organic compounds. Taking into account the high radioactivity level on primordial Earth, the ZnS edifices would have worked as natural converters of radioactive energy into reduced carbon and nitrogen-containing metabolites. The high-energy radiation could have penetrated deeper into the ZnS edifices than the solar UV light and, in addition, could have supported the ZnS photoreactors during the night hours.

Nonetheless, it is necessary to emphasize that the major source of energy for primordial life was solar light, since its energy, according to available estimates, was many orders of magnitude larger than that of ionizing radiation (see e.g. [19,20,34,109]). In addition, the unique *photo*stability of polynucleotides indicates that they were shaped by evolution under the selective pressure of solar light.

## Conclusion

Nurs'd by warm sun-beams in primeval caves

*Organic Life began beneath the waves*.

Erasmus Darwin, *The Temple of Nature*, 1802 [1]

The suggested Zn world scenario (i) identifies the geological conditions under which "primeval caves" made of photosynthesizing nanoparticles could emerge and persist on primordial Earth, (ii) comprises a mechanism by which these particles could use "warm sun-beams" for the production of diverse organic compounds, (iii) attributes the selection of primordial RNA-like polymers to their particular photostability, and (iv) indicates the driving forces and selective factors that might have promoted the transition from the first simple polymers to more complex living organisms.

The Zn world scenario shows how the "metabolism first" and "replication first" concepts can be reconciled and combined. The scenario comprises a mechanism of the continuous abiogenic photosynthesis of metabolites and their further conversion by ZnS-confined replicating entities.

The suggested concept invokes (photo)selection as a driving force – in the straightforward physical sense – in the emergence of increasingly complex biopolymers.

In addition, the Zn world scenario is detailed enough to enable falsification tests, as exemplified in the accompanying article [226].

Last but not least, the idea that life may have emerged within photosynthesizing, luminescent crystals – which gathered the light of the harsh Hadean Sun during the day and glittered through the nights – is aesthetically appealing.

## Competing interests

The author declares that he has no competing interests.

## Reviewers' Reports

### Reviewer 1

**Arcady R. Mushegian**, Stowers Institute for Medical Research, Kansas City, MO, and Department of Microbiology, Molecular Genetics, and Immunology, University of Kansas Medical Center, Kansas City, KS, USA

This is the first of two extremely interesting, thought-provoking manuscripts that discuss the hypothesis of the "Zn world" in which Life on Earth may have emerged. Both papers are elaborately argued and well (or, at times, beautifully) written.

The first paper leans heavily on (bio)physical and geological data, which is the area outside of my expertise. I have nominated Simon Silver to review it and have only brief remarks of my own. I have reviewed the second paper in much more detail.

p. 8 ln 14–17 "The evolutionary development from simplest prokaryotes to mammals is accompanied by a comparable increase in complexity, see e.g. [30,399]. This increase is routinely explained by Darwin's natural selection mechanism: more complex organisms emerge owing to their selective advantage over simpler predecessors [51,67,92,404]."

-- this whole discussion is not central to the theme, but, since the authors mentions it, there are certainly recent fundamental contributions to the topic that provide non-selective explanation for several crucial transitions in the process, most notably Michael Lynch's alternatives.

***Author's response***: *The sentence has been deleted upon the streamlining of the manuscript. Still I fully agree with this comment of the Reviewer*.

### Reviewer 1

p. 20 "It is noteworthy that if a protecting polypeptide could eventually discard excess energy by channeling it into a chemical reaction, then the probability of its own heat denaturation became lower; accordingly, the survival chances of the host replicator (that produced this peptide by using its own strand as a template) should increase."

-- replicator already able to translate RNA into peptides – is it not much later in evolution?

***Author's response***: *The phrase in parentheses has been deleted*.

### Reviewer 1

Manuscript as a whole: There is no discussion of the effect of various concentrations of the Zn ion on the stability of the phosphoester bond. (Fe^2+ ^ions are mentioned on pg 17 of the second paper, but not Zn^2+ ^ions). Even if the effect is negligible, this should be reviewed.

***Author's response***: *This is a very important point. Since Zn^2+ ^ions can catalyze the formation of proper 3'-5' linkages *[198,349], *they should also catalyze the cleavage of these linkages. Indeed, Zn^2+ ^ions have been shown to catalyze the cleavage of phosphoester bonds, particularly at high temperatures *[367,418,419]. *It is necessary to emphasize that the Zn world concept does not imply that primeval oligomerization was driven by Zn^2+ ^ions as one-way catalysts – these cannot exist. Instead, the Zn world concept suggests, following the ideas of Wächtershäuser *[124], *that the accumulation of primeval RNA-like oligonucleotides could proceed because it was thermodynamically favourable. This could be due to a combination of several factors, namely electrostatic matching, high atmospheric pressure, the potential to utilize the energy of light, photoselection, and so on. Therefore, Zn^2+ ^ions could accelerate the reactions but not affect their direction. However, the "life span" of primordial RNA-like polymers could have been increased if, after formation, they gained protection from the Zn^2+ ^catalyzed cleavage. In the revised manuscript, I discuss how the first polypeptides, by blocking the 2'-OH groups of ribose entities, may have prevented the binding of Zn^2+ ^ions to these groups and, hence, the chain cleavage*.

*The mechanism of polynucleotide cleavage by iron is quite different. Here, the predominant damage comes from harmful hydroxyl radicals that could be produced in the presence of redox-active Fe*^2+^/*Fe*^3+ ^*ions *[222,223,225]. *Because Fe*^2+ ^*ions are photoactive *[24,109], *the danger from hydroxyl radicals should be especially high in illuminated settings. Bearing in mind the expected high solubility of Fe*^2+ ^*ions in the primeval waters of 10*^-5^–*10*^-6 ^*M *[90,221,420], *we believe that Fe-rich, illuminated settings would have been hostile for pre-cellular life forms that, unlike modern organisms, had no protection from hydroxyl radicals (see also the accompanying article *[226]).

## Reviewer's report 2

**Simon Silver**, Department of Microbiology and Immunology, University of Illinois, Chicago, USA (nominated by Arcady Mushegian)

The two manuscripts by A.Y. Mulkidjanian and M. Galperin are a useful contribution to this long human effort (and a basic essential human need) to understand who we are and how we got here. The effort goes back at least to the ancient Greeks and with more modern developments starting with Louis Pasteur's demonstration of the absence of "spontaneous generation" of life in our time and then 75 years later A. I. Oparin taking some colloid physical chemistry and making for the first time a plausible scientific model of the Origin of Life. Then the Miller and Urey experiments (here in Chicago) with electrical sparks as energy showed one can synthesize small organic amino acids and other basic building blocks of biopolymers by abiogenic mechanisms, given the required energy.

There were three stages of the origin of life that should be defined better than Mulkidjanian does:

(a) Pre-biotic or abiotic, "pre-cellular" synthesis of building subunits of future biopolymers. This is not really the Origin of Life but something abiotic, a bit earlier and eventually leading to life, which starts with The First Cell. That is what is discussed in these papers. There are basically two current models for prebiotic small molecule organic chemistry and physical chemistry (which all agree is all that is needed) to progress to life. These can be divided roughly in to metabolism-first or self replication-starts-Darwinian evolution.

(b) The First Cell comes next and was (in our replication-oriented thinking) a ribozyme surrounded by a liposome (neither of which alone is alive). A source of external energy – undoubtedly metal cation redox derived – was the third requirement for the first cell [33]. Once the early Earth had these replicating primitive cells, Darwinian evolution and selection would lead inevitably to metabolism and the diversity of living cells we recognize today. The current authors disagree, but it is unclear whether the disagreement is fundamental or merely a matter of emphasis. This has been a field of multiple ideas but no consensus for most of the last 50 years. As one example lack of consensus, our 2005 paper seems to have had no influence on others thinking along alternative pathways. The First Cell lacked ribosomes, and therefore lacked proteins (e.g. The First Cell had no enzyme catalysts and no metabolism, as we understand now that these). It lacked ATP and other familiar high-energy phosphate-bond small intermediates. The early ZnS structures hypothesized by Mulkidjanian would be outside the First Cell, and might indeed contribute importantly to prebiotic accumulation of macromolecular precursors. However, once the primitive First Cell arose and evolution started, the cell membrane would block ZnS from the early cytoplasm.

(c) To go from The First Cell to The Last Universal Ancestor of Bacteria, Archaea, and then the first Eukaryotic cells (which formed by fusion of a couple or more prokaryotes) required evolution and invention of all of the familiar macromolecular nucleic acid and protein synthesis machinery and metabolism that is common to all current living cells, over the relatively brief period of a couple hundred million years, a short time back then over 3 billion years ago. The Genetic Code needed to evolve. Therefore Mulkidjanian is wrong on a small point that his ZnS photosynthetic mechanisms would do anything at all toward explaining how the three domains of life split, after basically all cell biology was invented by evolution. One should be clear and explicitly state that the "photosynthesis" referred to here is unrelated to biological photosynthesis, which came much later; early life was dependent, however, on metal ion redox chemistry; and was either utilized light (i.e. was photosynthetic) or not.

***Author's response***: *The three stages in the origin of life, as envisioned by the Reviewer, might serve as an instrumental model in some cases; the respective article *[33]*is cited in the revised manuscript. Generally, however, the evolution of life seems to be a more complex phenomenon. For example, viruses do not fit into this scheme at all. The very existence of viruses, which on the one hand are mostly built of nucleic acids and proteins but not lipids, and on the other hand seem to precede cellular life forms in evolution *[62], *challenges the idea of the First Cell that lacked proteins. This is one of the reasons why I base the Zn world hypothesis upon the "standard" sequence of events, namely RNA → proteins → membranes*.

*It seems plausible that the zinc-rich settings could directly affect the first life forms even after the emergence of membranes. The transition from membrane-lacking to membrane-encased life forms is connected with the following "chicken and egg" paradox: while a lipid membrane would be useless without membrane transporting systems, the respective membrane proteins would need membranes to evolve. We have tackled this paradox by combining structural and phylogenetic analyses and have suggested that the evolution of membrane transporters started from simple amphiphilic proteins capable of being incorporated into the membrane and of forming oligomeric pores and that chemiosmosis, particularly the proton-based type, was an evolutionary latecomer *[175,176,421]. *The porous membranes would still be leaky to Zn*^2+ ^*ions. We discuss this topic in a section devoted to the metallome of the Last Universal Common Ancestor of the accompanying article *[226].

*The mechanism to funnel the free energy of the Sun into the biosphere is envisioned as the abiogenic photosynthesis of diverse organic compounds that could then be processed by photoselected replicating entities. Accordingly, I suggest, following the views of Oparin *[3]* and Haldane *[5]* on this point, that the simple (bio)chemical processing of photosynthesized compounds (a kind of fermentation) could have preceded chemiosmosis. The relation between the abiogenic and biogenic photosyntheses is discussed in the accompanying article, in a section devoted to decline and fall of the Zinc world*. [226]. *We emphasize their fundamental similarity and argue that (bacterio)chlorophyll-based photosynthesis took over the function from ZnS-mediated photosynthesis*.

### Reviewer 2

It is nice to acknowledge here L.E. Orgel (1927–2007), who worked and thought productively and long on these questions. Orgel helped establish reluctantly and unintentionally that there never has been agreement of among the (accurately said by Mulkidjanian to be) "many scenarios" for the Origin of Life. It is implausible and unlikely to think that this new additional model of "A Zinc World" will add to or replace the currently popular RNA World [17] or that it will simplify and make the current alternatives fewer. Mulkidjanian is also wrong if he thinks one can experimentally "falsify" (as Karl Popper hopefully, but erroneously influenced a generation of mostly-British and German scientists) what happened nearly 4 billion years ago by current efforts mostly of analysis and argumentation, and not direct experimentation. The situation concerning understanding of the Origin of Life seems more like Thomas Kuhn taught us about other realms of science: ideas disappear only when the people who like them die and are replaced by younger people with differing ideas. A contemporary of L.E. Orgel, H. Morowitz together with younger colleagues [36] has a new popular report that lays out the issues nicely and also demonstrates that the current two efforts by Mulkidjanian and Galperin will not succeed in simplifying the alterative ideas. Trefil, Morowitz, and Smith [36] agree with Mulkidjanian and Galperin that metabolism came first, before hereditary polymer replication (this is where we primarily disagree) and they quote Albert Szent-Gyorgi (1893–1986) that Life is nothing but an electron looking for a place to rest. Redox chemistry and electron transport along pathways that conserve energy are essential to the Origin of Life as to contemporary life: on that we all agree. But the rest seems far indeed for consensus thinking.

There are major scientific groups and gatherings about these topics, so there really are "establishment views" (http://www.google.com is excellent and see the International Society for the Study of the Origin of Life and http://www.panspermia.org). But there is no consensus. There is instead wide range of interest and a wide range of ideas to which The Zn World is one more useful model.

***Author's response***: *The suggested concept of a Zn world complements the RNA world model by suggesting a physically and geologically plausible habitat for RNA-based life forms. Furthermore, the recent data of Sutherland and co-workers on the elevated photostability of natural nucleotides *[17]*support, in fact, the Zn world concept. It seems necessary to emphasize here that the "replication first" models, such as the RNA world, leave biologists happy but cannot completely satisfy those with a background in physics or physical chemistry who realize that RNA life would be thermodynamically implausible unless coupled to some utilizable energy source. In contrast, the "metabolism first" schemes are favoured by some physicists, chemists, and geologists, but these cannot convince the majority of biologists who simply cannot accept life without replication. By combining these currently duelling models, the Zn world concept may, hopefully, satisfy physicists, chemists, and geologists as well as biologists*.

*I am not the first to apply a Popperian approach to the origin of life problem (see e.g*. [58,227]). *This approach is based not only on falsification tests but also on experimentation. Therefore in the current article, I cite a wealth of experimental data in support of the suggestion that ZnS-mediated primordial photosynthesis could have powered the emergence of life. In the accompanying article *[226], *a separate section is devoted to the analysis of potentially feasible experimental tests of the Zn world concept*.

*I appreciate the reference to the review of Morowitz and co-workers *[36]; *this review is cited in the revised manuscript*.

## Reviewer 3

**Antoine Danchin**, Unit of Genetics of Bacterial Genomes, Department of the Genomes and Genetics, Institut Pasteur, Paris, France (Nominated by Eugene Koonin)

Photosynthetic origin of life in the Zn world, part 1, which I had to review is a compendium of two widely different topics.

A. This is a review article on multiple theories of the origin of life

B. This is a particular theory of a mineral origin of life, where Zinc containing minerals played a central role.

While I have only limited objections to part B (which I will detail below), I think that part A should be substantially modified to make a consistent piece of reflection on the origin of life.

Preliminary. The subject « origin of life » is not a standard scientific topic, as much of what can be proposed cannot be directly amenable to experimental approaches. In particular, even if we can produce plausible scenarios that would be short in time, the time lapses necessary to test them would be much longer than a human lifetime. This does not mean impossibility, but this implies that a fairly large section of what one can propose is of the domain of opinion rather than truth (to follow the Greek tradition). As we can only oppose an opinion to an opinion, my arguments will mainly be aiming at making the proposed work of higher quality, even when it will not fit my own opinion.

It is of interest to start from Erasmus Darwin, but to state that the problem of origin of life **started **with him is so inaccurate that it is really surprising that such a sentence could have been written. The author needs to say that he **chose **to start the question with Erasmus Darwin (presumably to make an amusing side-comment to the Darwin year).

***Author's response***: *In the revised manuscript, this sentence has been rewritten*.

### Reviewer 3

Immediately after this beginning, the author makes a very common erroneous confusion between reproduction and replication. While he quotes later on in the paper Freeman Dyson, he should have read (and followed) his demonstration that these are **not **at all the same, and that reproduction must have predated replication. This is, in fact, a major point for **any **scenario of the origin of life. I suspect, in fact, that quite a few of the references quoted in this « review » part of the paper have **not **been read carefully by the author. Perhaps should he restrict his references to papers he read in-depth. This would certainly make his review of models of scenarios of the origin of life less fuzzy and more accurate and incisive. In fact, rather than presenting a historical account of some scenarios (which may be interesting for philosophy of sciences, but not for a scientific article), the author should make a rational construct of all those theories. Once again, I think that the way things have been presented by Freeman Dyson is a model of what could (should) be done. This is a model of clarity.

Instead the author states: « We have argued that the "replication first" and "metabolism first" concepts complement rather than contradict each other and have suggested that the life on Earth started from a "metabolism-driven replication" ». I am afraid that this lacks proper rationality, and I would suggest him again to go back to Dyson, and re-think his scenario.

***Author's response***: *In the revised version of the article, this review section has been deleted. The section in which the Zn world concept was compared/related to other concepts on the origin of life has been somewhat expanded and moved to the end of the article. The relation between reproduction, metabolism, and replication is now considered in more depth*.

### Reviewer 3

Of course I would have liked to have also suggested the author to read my books " L'œuf et la poule, histoires du code génétique, Fayard 1983 – The Chicken and the Egg, stories of the genetic code " (translated into Japanese and Portuguese, not English) and « Une Aurore de Pierres. Aux origines de la vie, Le Seuil, 1990 – A dawn on stones. At the origins of life. » but it is in French (and translated into Portuguese, but not, unfortunately in English). Too bad, but this is life. So, I will have to summarise my arguments here.

***Author's response***: *The book Une Aurore de Pierres. Aux origines de la vie *[23]*proved to be very useful indeed and is now cited in the text*.

### Reviewer 3

The problem of the scenarios, as remarked by some authors (in particular Graham Cairns-Smith, in his Genetic Takeover) is that they have to decide about initial conditions. The soup cannot be a good one, as it is a poisonous soup (there is a need for selecting relevant compounds), and furthermore, as it lack essential components: coenzymes first (as they are the basis of metabolic pathways), then nucleotides (nucleobases are meaningless), basic amino acids, and long chain fatty acids, to chose a few of the essential ingredients. It must also **avoid **containing molecules that are too close to the ones retained later on. **No **scenario which does not take these constraints at their onset are or significant interest. Typically, the very fact that the author is not aware of these constraints (which, by the way, place metabolism first, for a reason complementary to that given in Dyson's demonstration) is shown in the fact that, for example, he writes about « adenine monophosphate » (p.10, l.5). It is absolutely not trivial to make a nucleotide, in particular because ribose is an extremely **unstable **molecule. And this implies a steady state process to make it...

***Author's response***: *"Adenine monophosphate" has been replaced by "adenosine monophosphate"*.

*Concerning the stability of nucleotides: Sutherland and co-workers have recently demonstrated the potential to make pyrimidine nucleotides from cyanamide, cyanoacetylene, glycolaldehyde, glyceraldehyde, and inorganic phosphate by circumventing the stages of single nucleobases and ribose *[17]. *Their synthesis yielded activated ribonucleotide β-ribocytidine-2',3'-cyclic phosphate as a major product and several co-products. Prolonged irradiation of this mixture by 254 nm light caused the destruction of various co-products and the partial conversion of ribonucleotide β-ribocytidine-2',3'-cyclic phosphate into ribonucleotide β-ribouridine-2',3'-cyclic phosphate. The authors concluded that there must be some (photo)protective mechanism functioning with the two natural nucleotides but not with other pyrimidine nucleosides and nucleotides. They write that "the mechanism provides a means whereby ...the two activated pyrimidine ribonucleotides needed for RNA synthesis, can be enriched relative to other end products of the assembly process" *[17]. *They have even claimed that "the prebiotic synthesis of activated pyrimidine nucleotides should be viewed as predisposed" *[17]. *This result is a clear-cut evidence that photoselection was a potential driving force behind the emergence of the first biological (macro)molecules*.

*Concerning the steady-state processes: I do not question their primacy. On the contrary, I argue that ZnS-mediated photosynthesis was the primary, steady-state process that supported the emergence of life. Unlike the postulated reactions at pyrite surfaces *[124], *which remain elusive, ZnS-mediated photosynthesis has been repeatedly shown to proceed with high yield at moderate temperatures *[144,149,272].

*However, I do not share the view of Dyson and others who consider the systems of interacting steady-state flows as alive. As we have argued elsewhere *[85], *it would seem more reasonable to date the origin of life to the establishment of the first replicating systems*.

### Reviewer 3

Now, a second part of any scenario, needs to state where we start, and from what data. If genetic takeover happened, then we are witnessing palimpsests (as suggested by Steve Benner) and there is not much hope, except for purely educated guesses. In contrast, if we still can have archives, then we could start from **extant **metabolism. This was the idea of Sam Granick and later on of Gunter Wächtershäuser (who ignored Granick, which made difficult uncovering his remarkable work: it took me several years to stumble upon that work). Here, the idea is to remark (which the author apparently ignores) that **anabolism **is very special in that it is essentially made of molecules carrying negative charges, often phosphate. Sulfur is also an essential component (as it remains for the author), associated to iron and to the idea of electron transfers (a neat idea with the prevailing conditions on Earth, when oxygen was not there, so that reversible electron transfers could be quite efficient).

***Author's response***: *Indeed, many metabolites carry negative charges, and we have extensively discussed this point in our previous article *[85]. *In the revised manuscript, it is now explicitly mentioned that photosynthetic reduction of CO_2 _yields negatively charged molecules that could remain confined to the ZnS surfaces*.

### Reviewer 3

Now, curiously (in fact I do not exactly understand why, as this would fit quite well with a model where zinc would play the role I think iron played) the author tries to discard the idea of surface metabolism. This is however, to my view, one of the most indisputable scenarios explaining how life could have led to a constructive metabolism. Granick, Wächtershäuser and I should include myself, argue very strongly for that step as the basis of **selection **(much needed to avoid the poisonous broth) and or **polymerisation**. True that Miller and De Duve (for reasons which are obscure to me, except that surfaces led to much less interest in sparks and the like) tried to discard the idea of surfaces by stating that chemical reactions on a surface would simply preclude separation from the surface of the polymers: this is a completely far-fetched argument, ignoring (purposedly?) that the polymerisation is not driven, in most cases by enthalpy, but by entropy (because of elimination of a water molecule, which goes in the solution: exactly the opposite of what would happen in the solution, where polymers would tend to be unstable unless the medium is highly compartmentalised, with a role on water activity). Their argument cannot be retained. Worse, I think that De Duve should have, on the contrary, built up his theory (which is excellent) of the role of thioesters, on surfaces: 4-phosphopantetheine would be the swinging arm to make many complex compounds **including the much wanted long fatty acids **!!! This is what I tried to demonstrate in my books (and in the short summary I published in English, with a conjecture on the synthesis of nucleotides [74]).

***Author's response***: *The idea of surface chemistry is not discarded. In contrast, I build upon the works of Wächtershäuser in this respect and argue that the syntheses of polymers could proceed at ZnS surfaces where the first RNA-like polymers were protected from photodamage. In the Zn world, therefore, the selection was not a one-step process but a two-step process: first, electrostatically preferred molecules adsorbed at the ZnS surfaces and could participate in diverse photochemical reactions, and next, those product molecules that were particularly photostable (e.g. oligonucleotides) were photoselected. As noted above, a photoselection of natural nucleotides has just been experimentally demonstrated by Sutherland and co-workers *[17]. *Thus, I consider the surface confinement of first polymers as a pre-condition of their survival in a UV-irradiated environment*.

*I do not think that it is possible to rebuff the argumentation of De Duve and Miller *[200], *because their reasoning was based on a straightforward application of energy conservation law. The mechanism of the membrane ATP synthase – as is considered in the article – exemplifies that, if the free energy (ΔG) of a particular synthetic reaction differs in two environments, then a portion of free energy – equal to the difference between the two respective ΔG values – should be "invested" to transfer the reaction product between the two environments. But, and this is the point that I have tried to emphasize, the displacements within the particular environment can proceed "for free". In other words, the polymers would not remain bound at the sites of their polymerization (as De Duve and Miller have envisioned), but rather could diffuse along the surface and interact with other polymers*.

### Reviewer 3

So much for these points: if I go on, I will write a new book! Just one final point: the author writes " The living organisms are evolving, dissipative systems that can exist only being supported by energy flow ", this is either trite or wrong (or both) and this rests on a completely wrong view of the way entropy fluxes are involved in Nature. The author (and I can send him the paper) should read the remarkable paper of Rolf Landauer " Inadequacy of entropy and entropy derivatives in characterizing the steady state " published in Physical Review A, in august 1975. He would certainly think twice, delete this sentence (which does not bring anything more than fuzzy words to his demonstration) and omit any reference to Prigogine anyway. This would not harm his work, quite the contrary.

***Author's response***: *It would seem that there is some misunderstanding on this point. If I understand properly, the Reviewer means that Prigogine was wrong while suggesting that dissipative structures tend to reach states with minimal entropy production. Landauer showed in his paper that this rule is not general and is valid only in some cases that are close to equilibrium *[422]. *The living systems are far from equilibrium, and therefore this rule is most likely not applicable to them in any case. I have been long aware of this controversy between Prigogine and his opponents, and therefore these ideas of Prigogine were not even mentioned in the original manuscript. I refer to Prigogine in another relation. Prigogine has repeatedly argued, in his many articles and books, that the emergence of ordered complexity in nature requires continuous supporting energy flow and out-of-equilibrium settings. Since some hypotheses on the origin of life simply ignore this point, I find it worthy to emphasize that life could not emerge either "for free" or in an equiponderated primordial soup – and cite Prigogine in this respect. Nevertheless, I have somewhat changed the respective wording to avoid any misinterpretations*.

### Reviewer 3

Now, I come to his opinion about zinc as central to the origin of life. This is based on an idea, worth investigating, placing light at the centre of energy management. Why not, this certainly happened with cyanobacteria and before. Why not very early on. I have no major objection to the generality of the idea. However I do not think that the way the author involves nucleobases is convincing. Light needs to be, indeed, coupled to energy, and electron transfers are cases in point. This may place heme or related molecules in the limelight, but also ... iron. Certainly not nucleic acids. In contrast, I would certainly accept ZnS (but I did not have access to the second paper, where falsification tests are proposed). Photosynthesis in a Zn world, why not, and this apparently would happen as a mineral surface metabolism (so that I did not really understand why the author was so much against the idea). But I would not easily accept nucleobases as primitive: as I stated above I think nucleotides must be made more or less directly, and I proposed a scenario which sees them as a leak of a general process of nitrogen fixation. This might well work in a Zn world.

***Author's response***: *While being quite happy that the Reviewer has no major objection to the idea of abiogenic ZnS-mediated photosynthesis, I would like to reiterate that I am not against the idea of surface "metabolism" (see above)*.

*I readily agree that it is difficult even to imagine a spontaneous formation of nucleotides in the "primeval broth" from occasionally formed nucleobases, ribose molecules, and phosphate groups. The Zn world concept, however, does not require the formation of single nucleotides in solution. The concept implies that polynucleotides could directly assemble at the ZnS surfaces from simpler building blocks. In the aforementioned work of Sutherland and co-workers, the viability of such syntheses has been experimentally demonstrated *[17]. *In this way, the step of mononucleotide synthesis could be bypassed. The survival of the entire polymeric constructs could then be guaranteed by their extreme photostability. In this framework, single nucleotides could have first appeared as cleavage products of the polynucleotides*.

### Reviewer 3

Second, I would urge the author to think of replication as secondary to metabolic reproduction, where peptides were present first (this is what I tried to demonstrate in my "The chicken and the egg" book (see above)). Also, the author, once again, needs to think of the origin of coenzymes! This can certainly be included in his scenario. Nucleic acids are latecomers, they came first as surface substitutes, then discovered that rather than being **substrates **they could become **templates**. There I am interested by the idea that Zn favors formation of 3'-5' bonds (which is indeed a very difficult feat, normally favoured by the presence of peptides).

***Author's response***: *In fact, I think (and write) about replication as a phenomenon that was secondary to the reproducing, steady-state flows of energy and matter (although I think the word "metabolism" might be misleading in relation to abiogenic processes)*.

*The "metabolism first" models, however, have certain difficulties in explaining the transition to the first replicating entities. For example, Dyson has suggested a multistep model with the reproducing but not replicating cells emerging first and followed by nucleotide-based replicators initially as parasites of these cells and then as their symbionts *[398]. *However, neither the (bio)chemical origin of the replicators nor the selection factors that could favour their emergence were addressed. In contrast, the Zn world model considers photoselection as a force that, by driving the emergence of photostable entities built of stacked and paired nucleotides, could have paved the way to the first self-replicating systems*.

*Therefore, I cannot agree that nucleic acids were latecomers. In the literature, many arguments are in favour of RNA-first scenarios, the majority referring to the ability of RNA molecules, but not proteins, to replicate; I do not want to repeat this argumentation here. One more argument, based on selection criteria, can still be invoked. One can presume that nucleobases, as well as amino acids, could be selected for their particular tasks because of specific properties that were useful under given circumstances and were shared by all nucleobases and amino acids, respectively. The common property of nucleobases is their extreme photostability. Hence it has been repeatedly hypothesized that they were selected because of this property (see *[17,85,159,165]* and references therein). The respective selective factor, solar UV irradiation, is abiotic, so that the selection could have started before the origin of life. The common property of amino acids is the presence of amino and acidic groups that allow them to join into polypeptide chains. The selection for this property, however, implies the existence of devices that could connect amino acids by peptide bonds. In modern organisms, this function is routinely performed by RNA-based machines (ribosomes); it is even now becoming clear how these machines could evolve from simpler RNA constructs already capable of peptide bond formation (see *[73]* and references cited therein). This consideration, in my opinion, strongly supports the RNA-first schemes*.

### Reviewer 3

Finally, a word about the quotation by the author of the work of Rolf Landauer. It happens that I was lucky enough to meet him before he passed away, and to discuss much of the questions involving entropy and information (he showed to me how Prigogine was completely wrong, for example, see his paper cited above). The important point here is to see that if creation of information is not a real problem, accumulation of information is. And this requires an energy source. It is most likely, to my view, that this was originally performed by a mineral such as polyphosphate, which is quite metastable, and later on perhaps by nucleotides and polynucleotides (such as what is seen in the management of energy in the degradosome today, see my Phylogenetic view of ribonucleases [423]).

***Author's response***: *The energetics of the accumulation of information is a very important point. Since polynucleotides are not eternal, replication per se can be considered as a means to preserve (accumulate) information before an information carrier – a polynucleotide strand – eventually breaks down because of thermal fluctuation or (photo)cleavage. In the manuscript, several tentative energy sources for replication are considered. Initially, the replication could be supported by the thermodynamically coupled cleavage of available polynucleotides – "failed" information carriers, in the given context. The cleavage of inorganic polyphosphates, as suggested by the Reviewer, could provide energy as well; chemically, this reaction is similar to the cleavage of polynucleotides. This possibility is now mentioned*.

### Reviewer 3

Well. To complete this review, I think that the author should reorganise it completely, shorten some of his points and take into account really seriously the reflection of Freeman Dyson, which he places only at the end of his text.

Of course as much of what I wrote is opinion (doxa), I have no strong reluctance for the publication of this work.

***Author's response***: *The text has been streamlined, and the discussion of Dyson's views has been expanded. In the revised manuscript, photoselection is explicitly considered as a possible bridge between reproduction and replication*.

## Reviewer 4

**Dieter Braun**, Systems Biophysics, Functional Nanosystems, Ludwig Maximilians University Munich, München, Germany (Nominated by Sergey Maslov)

While I am sympathetic to the core idea presented, I cannot support the manuscript at this stage for publication. This decision stems from a number of questions I would have liked to have answered and, to a considerable degree, to the vast length of the manuscript. The amount of novel information by far does not warrant the length of the manuscript, which, together with the second part, is rather a book manuscript than a scientific paper. The strain put on both the reader and the referee by the length is not acceptable.

I would like to urge to shorten the manuscript to at least half of its length by omitting review-like passages, rather vague ideas and a large amount of repetitions in the text. I will indicate sections which I consider not very helpful as I will walk through the text. Being an experimental biophysicist, I am not used nor happy to adapt to novelistic manuscript sizes for the publication of a mere hypothesis. I only see the need for exceptions for detailed experimental results or detailed theoretical treatments. But even without paper publishing, there is the need for shortness due to time limitations of the readers (and the referee). I could not check enough references well enough as I usually like to do due to their sheer number.

***Author's response***:

*Being an experimental biophysicist myself, I usually use a more laconic style while describing experimental results, see e.g*. [424]. *The standard style, however, is not wholly applicable to a hypothesis paper on the origin of life. Nonetheless, I have attempted to address the concerns of the Reviewer wherever possible*.

### Reviewer 4

First, for a hypothesis paper, the manuscript does a good job in collecting literature for a specific scenario, namely UV-triggered biosynthesis on Zn-surfaces. Also the focus on possible physical boundary conditions is the way to go in origin of life research in my viewpoint. The paper's discussion will lead to new experiments, which is a good thing.

That said, I think the author is a bit 'pushy' on his proposal – calling it Zn-World as if Zinc would be a common biological molecular construction. Also saying in the introduction that the Zn world proposal "... resolves the conflict between the "metabolism first" and the "replication first" concepts of abiogenesis, which are suggested to reflect the two facets of the Zn world, namely the continuous abiogenic photosynthesis of metabolites and their further conversion by the ZnS-confined replicating entities." may be correct on the first, but sketchy to say the least on the second.

***Author's response***: *The tag "Zn world" was chosen to emphasize that the (photo)chemical properties of Zn^2+ ^ions and Zn-containing substances could have shaped the first life. While the photochemistry of ZnS crystals could have governed the nature of photosynthesized compounds and that of their photo-derivates, the catalytic properties of Zn^2+ ^ions may have determined the particular traits of the first (bio)polymers, e.g. the choice of 3'-5' linkages for RNA strands*.

### Reviewer 4

Why the author chooses to make far-fetched connections to very old literature (Erasmus, Darwin's pond, to give two examples), I do not understand. Literature from times where the molecular basis of biology or thermodynamics was not understood can only be lucky to make a proper proposal, but is not helpful to be repeated in current literature.

***Author's response***: *I quote the older literature to express my deep respect for scientists who could make suggestions that were correct from the viewpoint of thermodynamics in "times where the molecular basis of biology or thermodynamics was not understood". Ironically, some hypotheses on the origin of life that were put forward during the last decades appear shaky from the thermodynamic viewpoint*.

### Reviewer 4

I am missing a discussion on whether clouds have obscured early earth or whether land mass and shallow waters have existed at all on the early earth. This would substantiate two fundamentals of the concept.

***Author's response***: *Clouds, if there were any at that time, should have consisted of water. Water is transparent to UV beams (of wavelength > 200 nm *[425]). *Therefore, one can become tanned even on a cloudy day*.

*The presence of the first continents already at 4.2 Ga *[188]*implies a shoreline around them and water patches at their surface, in particular at sites of volcanic or geothermal activity*.

### Reviewer 4

Another question I am missing in the manuscript is in which geometry and location the author sees the UV radiation not being absorbed by porous rock or sedimentation layers. As it stands, the argument is basically restricted to surface water some meters deep and rock structures some micrometer thin.

***Author's response***: *Taking into account the expected interplay between the continuous precipitation of ZnS and photocorrosion, I would instead estimate the thickness of the habitable layer as being several millimetres (similar to that of modern microbial mats built up of phototrophic prokaryotes)*.

*In addition, I would like to note that the ZnS-covered areas could spread over hundreds of square kilometres – owing to the high thermal activity of the young Earth*.

### Reviewer 4

Above relates to the puzzle I have how a Zn world should accumulate and trap their molecules by a nonequilibrium process and at the same time be fully exposed to UV radiation. I guess, exposure to UV inherently means that the molecules synthesized diffuse then out into the open waters?

***Author's response***: *As noted in the revised manuscript, the photosynthesized molecules should be electrically charged and could adsorb at the surface of ZnS (see also *[85]). *In addition, the porous structure of ZnS precipitates should constrain the diffusion of photosynthesized molecules out into the open waters (see the structure of modern ZnS precipitates in *[179-181]). *Moreover, as discussed in relation to the works of Wächtershäuser *[124,126], *the molecules that were polymerized at the surface would remain confined to the surface*.

### Reviewer 4

The author takes "Mauserall has introduced a major additional constraint by noting that the energy requirements of the first living beings had to be compatible with that of modern organisms [109]. He argued that "the ur-cell would be simpler, but it would also be less efficient" " to the conclusion "More rigorously speaking, the intensity of the energy flux(es) that supported the emergence of life should be either comparable with the intensity of modern life-supporting energy flows or even stronger.". I do not see such a strict logic connection, since it assumes that the biomass production should have been equal. It would be helpful here if the authors would clarify his understanding of "intensity" (i.e. number of photons) as opposed to for example "wavelength" (i.e. the energy gap).

***Author's response***: *Here I would just like to quote David Mauzerall: "The ur-cell would be simpler, but it would also be less efficient. A crude number for this energy flux is 10 mwatts per gram of biocarbon. In anticipation of the stress on photochemistry, this flux can be normalized to energy flux per area, using the area of a 'typical' prokaryote cell ~10 μ^2 ^and its weight of carbon, 0.1 ng. This number is 0.01 mwatt per cm^2^*. Table 1 s*hows the energy sources of the primitive earth. It is clear that only solar photon energy is sufficient for the continuing evolution of life." The quotation is taken from *[109], *in which the table referred to can be found*.

### Reviewer 4

At the core of the argument is the finding that DNA/RNA can get rid of harder light quanta by the hydrogen bonding of the bases. However why then are the bases constructed such that they absorb UV radiation with good efficiency at all? The absorption cross section is so large that it is even used for DNA quantification. I would guess that one could probably readily design molecules which would combine both a lower UV absorption and – if they do still absorb one – get rid of the energy with similar mechanisms. Am I missing something here?

***Author's response***: *Lower UV absorption of a chemical compound means that a passing UV quantum is captured not by the first molecule on its way but, say, by the tenth molecule. But then this tenth molecule still has to cope with the entire energy of the UV quantum. As long as organic molecules are considered, deactivation of a UV quantum requires prompt spreading of its energy over many bonds (therefore conjugated bonds are needed) and an efficient sink for energy (here several diverse mechanisms might be involved *[164,165]). *In the absence of conjugated bonds, the energy of UV quanta will "gather" at the "weakest" bond and eventually break it. As a result, the quantum yield of photodamage might be much higher for a compound with low absorption of UV than for a compound with high UV absorption and many conjugated bonds. Goosen and Kloosterboer *[166]*have shown that the release of phosphate in response to 254 nm light was 10 times slower in the case of adenosine monophosphate (AMP, a highly UV-absorbing compound) than in the case of glycerol 2-phosphate (which barely absorbs at 254 nm). It would seem that, in a kind of trade-off, the increased absorption cross section was over-compensated by increased photostabilty in the case of AMP. In this framework, all means that increased the spreading of excitation energy without increasing the absorption cross section would be strongly favoured by evolution. The stacking of nucleotides, as well as their pairing, would result in the spreading of excitation energy over a larger number of chemical bonds without increasing the absorption cross section*.

### Reviewer 4

It would be useful if the author could also comment on the "Lost City" type vents as his arguments on page 10 apparently only applies to so called "black smokers".

***Author's response***: *Lost City-type vents were found at depths of ~700–800 m (see *[102]*for a review). Their hydrothermal fluids were cooler than those of black and white smokers (45°C–75°C versus 250°C-350°C). Not surprisingly, these fluids were depleted of any transition metals and were low in H*_2_*S. It is not clear how metabolism of any type could emerge under such conditions in the absence of transition metals as catalysts*.

### Reviewer 4

Beginning with about page 15 the manuscript tends to become highly repetitive or very vague in its argumentation. I would like to point to "low density" parts of the manuscript in the following. Shortening there would make the manuscript much more accessible.

The repetitive sections of the manuscript start at page 15. I will give a short list where I see strong repetitions:

- 15.22–16.2: Spreading excitations were mentioned before

- 16.3–16.11: highly repetitive

- 22.17–23.15: I do not see why the paper needs to cite Popper here.

- 23.18–24.28: I see this as repetitive review of thoughts formulated before

- 25.23–27.15: Many repetitions, things of review character and not adding much to the hypothesis proposed

- 27.16–28.8.: Highly repetitive

- 29.21–29.27 was mentioned before

- 34.20–35.13 is quite repetitive

- 37.4–37.11 was mentioned before

- 39.3–40.15: I do not understand why we need a review of Oparin and Haldane here.

- 42.13–46.14 is both repetitive and vague

- 46.23–47.18 was said before quite often

- 48.23–49.16 is a review and does not add much to the discussion

- 50.5–50.12 is repetitive

***Author's response***: *Hopefully, the repetitions are less frequent in the revised manuscript*.

### Reviewer 4

There are rather vague ideas:

- 17.7–17.17: The conclusion that dsRNA-selection leads to replications systems is not well documented.

***Author's response***: *Regretfully, I cannot document better the properties of the first replicating entities that emerged more than 3.5 Ga ago. However, the self-replicating RNA system, as presented recently by Lincoln and Joyce *[72], *consists of ribozymes that are built of paired RNA strands*.

### Reviewer 4

- 18.1–18.12: The should-heating of a hundred 'unit' RNA-like polymer should be complemented by more data, for example by the time scale and photon rate over which the tens of degrees are applied. I would think there is a difference between bleaching and thermal damage. The subsequent conclusions in the manuscript are in my view far fetched.

***Author's response***: *The estimate from *[210], *which I have used, was calculated for an ensemble of bacteriorhodopsin molecules, each being hit by one quantum of visible light. I would strongly encourage a corresponding quantitative analysis for RNA molecules and UV light quanta*.

### Reviewer 4

- 18.19–22.15: Much of what is written here is rather hopeful speculation and uses imprecise language.

***Author's response***: *Indeed, the story of how the first proteins could have emerged is hopeful speculation. However, the field is developing fast. In the revised manuscript, I invoke the just-published seminal work of Bokov and Steinberg *[73]* and use more precise language*.

### Reviewer 4

- 33.23–34.2: I cannot see the biological evidence which supports the connection between dye sensitization on ZnS surfaces with "zyms" helping in adding the yield of metabolite production.

***Author's response***: *Although the photochemical interactions between polynucleotides and ZnS/CdS quantum dots have been actively studied, the light-gathering function of polynucleotides in such systems, to the best of my knowledge, has not yet been quantified. Therefore, I have used as my basis quantitative data obtained with ZnS crystals and adsorbed organic dyes *[329].

### Reviewer 4

- 37.20–37.24: It is not clear to me what colonization waves have to do with the "Zinc" world.

***Author's response***: *From a biological viewpoint, the transition from the Zn world to modern life has to be envisioned in an evolutionary scenario*.

### Reviewer 4

- 48.4–48.22: Both is the hypothesis presented here vague and I also do not see a connection to the Zn-World.

***Author's response***: *The Zn world concept has two major constituents, namely ZnS-catalyzed photosynthesis and the UV selection of most photostable polymers *[112]. *Accordingly, the emergence of the first information-carrying polymers as a result of photoselection is a legitimate topic of the Zn world scenario*.

### Reviewer 4

Also, a strange reference is "in accordance with the "rings joined to rings" scenario of Erasmus " on page 16.4 which can hardly be taken seriously.

***Author's response***: *In my opinion, the expression "rings joined to rings", taken from the work of Erasmus Darwin *[1], *adequately describes the emergence of the first RNA-like polymers. Their formation should have invoked both the stacking of ring-like nucleobases and their Watson-Crick pairing*.

### Reviewer 4

Much more detail would be in my opinion necessary to relate the presented scheme of UV absorption to X-rays. Much more reaction surface could be gained at the subsurface. However we only read "Taking into account the high radiation level ... converters of radioactive energy into reduced carbon and nitrogen containing metabolites." Some more elaboration on this point would be very helpful.

***Author's response***: *Radiation chemistry is not my field of expertise. A more detailed analysis of the "radio-synthesis" of organic compounds within ZnS edifices might be a worthwhile task*.

### Reviewer 4

Last but not least I find the last sentence of the paper rather revealing: what could the aesthetics of minerals to do with a scientific argument on the origin of life?

***Author's response***: *Aesthetic criteria are of great importance in scientific research, see e.g*. [426]. *For example, my initial opposition to the idea of abiogenesis at the floor of the Hadean ocean *[117], *when I first heard about it, was purely aesthetic. I simply did not like the idea of the origin of life being in complete darkness. Only later on I realized the (bio)chemical shortages of this scenario and, at the same time, learned to appreciate the seminal idea of Russell and co-workers on inorganic metal-sulfide compartments as incubators for the first life forms. As a result, initial aesthetic opposition could be transformed into a scientific context*.

### Reviewer 4

I know that the open refereeing process makes it risky to raise the voice (the scientific exchange has many possible for punishment) – but without accepting criticism there is no progress in science.

***Author's response***: *I fully agree with this statement by the Reviewer*. *A major advantage of the open review procedure is that referees are free to express their own opinions on the subject. As a result, the open exchange of ideas between authors and reviewers has its own value*.
